# Supplementary material for: Knee moments of anterior cruciate ligament reconstructed and control participants during normal and inclined walking
Source: BMJ Open. 2014 Jun 4;4(6):e004753. doi: 10.1136/bmjopen-2013-004753 (PMC4054639; doi:10.1136/bmjopen-2013-004753)
Supplement: Author's manuscript [file bmjopen-2013-004753.draft_revisions.pdf]

Knee moments of anterior cruciate ligament reconstructed and control participants during normal and inclined walking.

Journal:	*BMJ Open*
Manuscript ID:	bmjopen-2013-004753
Article Type:	Research
Date Submitted by the Author:	24-Dec-2013
Complete List of Authors:	Varma, Raghav; Imperial College London, MSK Lab<br>Duffell, Lynsey; Imperial College London, MSK Lab<br>Nathwani, Dinesh; Imperial College London, MSK Lab<br>McGregor, Alison; Imperial College London, MSK Lab
**Primary Subject Heading**:	Sports and exercise medicine
Secondary Subject Heading:	Rehabilitation medicine
Keywords:	Knee < ORTHOPAEDIC & TRAUMA SURGERY, Orthopaedic sports trauma < ORTHOPAEDIC & TRAUMA SURGERY, SPORTS MEDICINE

SCHOLARONE™
Manuscripts

For peer review only

Title: Knee moments of anterior cruciate ligament reconstructed and control participants during normal and inclined walking.

Authors: Raghav K Varma[1], Lynsey D Duffell[1], Dinesh Nathwani[1] & Alison H McGregor [1].

Affiliations: 1) MSK Lab, Imperial College London, UK

Corresponding Author:

Professor Alison McGregor

MSK Lab

Imperial College London

Floor 7, Laboratory Block

Charing Cross Hospital, Fulham Palace Road

London, W6 8RP

T: 020 3313 8834

F: 020 3313 8835

E: a.mcgregor@imperial.ac.uk

Word count: 2,460

Key words: Anterior cruciate ligament reconstruction, inclined walkway, adduction moment and gait analysis.

For peer review only

Abstract

Background

Prior injury to the knee, particularly anterior cruciate ligament (ACL) injury is known to predispose to premature osteoarthritis (OA). The study sought to explore if there was a biomechanical rationale for this process by investigating changes to knee function during routine daily activities.

Methods

Twelve subjects who had undergone ACL reconstruction (ACLR) and 12 volunteers with no history of knee trauma or injury were recruited into this study. Gait was assessed during flat (normal and slow speed), and uphill and downhill walking using a bespoke inclined walkway with an embedded Kistler Force plate (Kistler Instrumented AG, Winterthur, Switzerland), and a ten camera Vicon motion capture system.

Results

No significant difference was observed in the peak knee adduction moment between ACLR and control participants however, in further analysis, a one way ANOVA revealed that ACLR participants with an additional meniscal tear or collateral ligament damage (7 subjects) had a significantly higher adduction moment (0.33 ± 0.12 Nm/kg.m) when compared to those with isolated ACLR (5 subjects, 0.1 ± 0.057 Nm/kg.m) during normal gait ($p<0.05$). A similar, but non- significant trend was seen in all the other activities.

For peer review only - http://bmjopen.bmj.com/site/about/guidelines.xhtml

Conclusion

Subjects with an isolated ACLR had a reduced adductor moment rather an increased moment, thus questioning prior theories on OA development. In contrast those subjects who had sustained associated trauma to other key knee structures were observed to have an increased adduction moment. This fits with the theory that it is additional injury following ACL rupture that has a higher predisposition to osteoarthritis rather than isolated ACL deficiency alone.

For peer review only

Article summary

Strengths and limitations of this study

- To our knowledge this is the first report looking at external moments during inclined and declined walking for ACL reconstruction participants.
- In addition to looking into the external moments of the affected and unaffected knee, this study also looked at the effect of gait speed on external moments and differences in knee osteoarthritis outcome score (KOOS) for each group.
- This study provides an explanation for the disparity seen in previous studies looking into peak knee adduction moment in ACLR and matched control subjects.
- This study suggests that injuries to other key knee structures may play a bigger part in inducing osteoarthritis than ACL injury alone.
- One limitation to this study is the small sample size, in particular after dividing our ACLR group into ACLR + and ACLR – groups.

For peer review only - http://bmjopen.bmj.com/site/about/guidelines.xhtml

Introduction

Anterior Cruciate Ligament (ACL) injuries are common, exceeding 100,000 annual cases in the United States.(1) The majority are sports related injuries, and lead to knee instability as a result of increased anterior tibial translation and anterolateral rotation.(2) ACL reconstruction (ACLR) is the primary treatment for an ACL rupture and permits return to a range of high-level activities including sport. It is accepted that people with ACL injuries, including those who undergo surgical reconstruction, are prone to further knee degeneration(3) and early OA.(4,5) Lohmander et al. (2007) reviewed 127 publications and determined that the overall mean incidence of developing OA after an ACL injury with/without reconstruction to be over 50%(3) with the majority noting radiographic signs of OA 10 years after injury.(3) Gait biomechanics are considered to play a vital part in knee joint degeneration, (5, 6) with altered kinematics and kinetics changing the distribution of mechanical load on the knee. (7) This in turn is postulated to lead to cartilage wear(5 - 7) and eventually knee osteoarthritis.

There is consensus amongst researchers that ACL deficient patients employ different gait strategies.(8) In vivo studies have found reduced knee flexion,(5) increased internal tibial rotation(5, 9) and increased knee adduction moment(10) during level walking, to be the three main changes in external knee moments following an ACL rupture. Furthermore, research has indicated that ACL reconstruction does not restore normal knee mechanics. Berchuck et al. in 1990(11) noted reduced knee flexion during normal gait indicating a coping strategy called quadriceps avoidance gait. Anterior displacement of tibia through the contraction of quadriceps is balanced by the ACL when the knee is at an angle of 0 to 45 degrees.(11) People with ACL rupture and/or reconstruction may

For peer review only - http://bmjopen.bmj.com/site/about/guidelines.xhtml

involuntarily avoid quadriceps activation(12) to prevent further ACL damage. Gait adaptations in the sagittal plane can lead to knee joint instability and ligament laxity.(13) This may result in osteoarthritis initiation and progression.(13)

High moments in frontal and transverse planes of the knee have been linked to OA.(5) ACLR has been shown to restore rotational stability,(9, 14) however high knee adduction moments (KAM) after reconstruction have been observed (15) but such changes are not universally agreed(16, 17) This is of importance since a 1% increase in adduction of moment at the knee is thought to increase the risk of knee OA by 6.5 times.(18) Hence in this study we aim to gain a better understanding of peak knee moments in the sagittal and frontal plane. Our primary aim is to compare peak knee moments in the sagittal and frontal plane of ACLR participants to healthy controls on sloped surfaces. We additionally investigated the effect of speed on peak moments.

Methods

This cross-sectional study explored peak knee moments between ACLR and healthy control subjects during slope walking. The study was approved by Imperial College Research Ethics Committee.

A total number of 24 subjects participated in this study and written informed consent obtained, details are provided in Table 1; 12 (9 male and 3 female) ACLR participants (age, 24.83 ± 8.81 year; weight 75 ± 11.13 kg; height 1.76 ± 0.13 m); and 12 (9 male and 3 female) control participants (age, 30.5 ± 8.68 year; weight 71.6 ± 11.2 kg; height 1.73 ± 0.11). The ACLR inclusion criteria were; aged between 18 – 60 years, Body mass

For peer review only - http://bmjopen.bmj.com/site/about/guidelines.xhtml

index (BMI) < 30kg/m^2, a complete, unilateral ACL rupture followed by a single bundle hamstring auto graft reconstruction that was performed at least one-year ago, no history of knee trauma or injury to their contralateral leg. Subjects who were unable to walk comfortably on a 10-degree incline walkway were also excluded. The control group did not have any muscular or neurological lower limb pathology and were matched to the ACLR subjects with respect to height, weight and their dominant leg (leg preference for kicking). All subject completed the Knee injury and Osteoarthritis Outcome Score (KOOS)(19).

A three-dimensional motion analysis system (Vicon MX™ T-20 System, Vicon, Oxford) was used to collect kinematic data for normal, slow, upslope and downslope gait. This software used 10 motion capture cameras to pick up 35 reflective markers at a sampling rate of 100Hz. The reflective markers were placed bilaterally on the head of 2nd metatarsal, head of 5th metatarsal, head of 2nd tarsal, calcaneal tuberosity, medial and lateral malleolus, medial and lateral femoral epicondyle, anterior superior iliac spine, posterior superior iliac spine, acromion and one single marker on the manubrium. Marker clusters were affixed bilaterally to the calf and thigh. Kinetic data (ground reaction force) were collected using portable force plates (Kistler Instruments AG, Winterthur, Switzerland) at a sampling rate of 1000 Hz and was synchronised with the camera data.

The subjects were asked to walk barefoot. A 5 minute self-directed warm up allowed the subjects to familiarise themselves with each task.

For peer review only - http://bmjopen.bmj.com/site/about/guidelines.xhtml

A 7-meter long walkway was used, 2.5-meters of which could be raised to form a ramp, at an incline of 10 degrees. It was constructed from a steel frame and covered with plywood, with one portable force plate embedded in the centre (Figure 1). Participants were asked to walk at a self-selected pace both uphill and downhill. The ramp was then removed to create a level walkway. All the participants were asked to walk at a self-selected pace and at a pace, which they consider to be slow. Each task was repeated until both feet made complete contact with the middle of the force plate at least three times.

All data was time normalised to one gait cycle. The data was analysed from the stance phase of the gait cycle; when the ground reaction force reached more than 40N (heel strike) to when it dropped to less than 40N (toe off). A fourth order Butterworth Filter at a cut off (12 Hz) was used to reduce noise. Joint angles and moments were calculated from the position of the reflective markers and the ground reaction force data using a custom model written in body builder software.(20-22) Peak moments in the sagittal and frontal planes of the knee were extracted using MATLAB (R2013b) software.

Paired Student's t-tests were used to determine any significant differences in demographics between ACLR and control group. A one-Way ANOVA was used to calculate significant differences in all other parameters. Holm-Sidak approach was used to establish significance, with the alpha value set at 0.05. All statistics were carried out using SigmaPlot 11.0 on Windows.

For peer review only

Results

The ACLR group was significantly older than the control group ($p<0.05$). A multiple linear regression was carried out which showed that age ($p = 0.65$) did not have a significant effect on ACLR peak adduction moment. There were no significant differences between groups in height, weight and Tegner Activity Scale (Table 1). The mean time since reconstruction surgery was 4 years and 6 months.

Table 1 Subject characteristics, activity level and time since surgery. 12 participants in ACLR group and 12 in control group

	ACLR (SD)	Control (SD)	Paired T-test
Age (yr)	30.5 (8.68)	24.8 (8.81)	P = 0.011
Height (m)	1.76 (0.13)	1.73 (0.11)	P = 0.078
Weight (kg)	75 (11.13)	71.6 (11.2)	P = 0.535
Tegner Activity Scale	6.25 (1.82)	6.08 (1.93)	P = 0.755
Time since surgery (yr)	4.5 (3.5)	NA	

ACLR, Anterior cruciate ligament reconstruction

We further divided our ACLR group into two; subjects that had additional cartilage, meniscus or ligament damage in their ACLR leg (ACLR + group; 7 subjects) and subjects with isolated ACL injuries (ACLR – group; 5 subjects). The additional knee injuries to the ACL rupture are meniscal tear (3 participants), cartilage damage (3 participants) and torn MCL (1 participant).

The control group had significantly higher scores in all of the KOOS domains apart from activities of daily life compared to the ACLR group (Table 2). No significant KOOS differences were seen between ACLR + and ACLR – groups. Table 3 shows that there were no significant differences in gait speed in normal or slow walking between any groups.

For peer review only - http://bmjopen.bmj.com/site/about/guidelines.xhtml

Table 2 Knee osteoarthritis outcome score (KOOS) with standard deviation (SD) for each domain recorded for each group.

KOOS Outcome	ACLR (SD) (n=12)	Control (SD) (n=12)	ACLR + (SD) (n=7)	ACLR - (SD) (n=5)	P-value	Post-hoc analysis
Pain	88.4 (9.32)	99.1 (3.2)	87.5 (8.83)	89.4 (10.8)	P = 0.006	Control vs ACLR; Control vs ACLR +
Symptoms	83.1 (11.4)	98.2 (3.19)	85.1 (12.7)	80.7 (10.6)	P = 0.001	Control vs all other groups
Activities of daily life	96.3 (5.63)	100 (0)	98 (3)	94.4 (7.7)	P = 0.090	
Sport and recreation	83.8 (16.9)	99.6 (1.4)	89.1 (7.4)	77.4 (23)	P = 0.010	Control vs ACLR; Control vs ACLR -
Knee related QOL	64.5 (23.2)	100 (0)	64.6 (23.3)	70 (26.7)	P = 0.001	Control vs all other groups

ACLR, Anterior cruciate ligament reconstruction; ACLR +, subjects with other knee injuries in their ACLR leg; ACLR -, subjects with isolated ACL injuries.
Data are mean (SD).

Table 3 Gait speed during normal and slow, level walking tasks.

	ACLR	Control	ACLR +	ACLR -	P = value
Gait normal speed	1.17 (0.13)	1.20 (0.11)	1.18 (0.15)	1.16 (0.11)	P = 0.916
Gait slow speed	0.76 (0.13)	0.75 (0.11)	0.78 (0.16)	0.74 (0.09)	P = 0.946

ACLR, Anterior cruciate ligament reconstruction; ACLR +, subjects with other knee injuries in their ACLR leg; ACLR -, subjects with isolated ACL injuries.
Data are mean (SD).

No statistically significant differences were found in peak knee adduction moment between ACLR and control participants during normal, slow, uphill and downhill gait.

For peer review only

Further analysis revealed that ACLR participants with meniscal tear, cartilage damage or medial collateral ligament damage (ACLR +) had significantly higher knee adduction moment (0.33 ± 0.12 Nm/kg.m) during normal gait compared to those participants with an isolated ACL injury (ACLR -, 0.1 ± 0.057 Nm/kg.m), p = 0.042 (Figure 2).

This was not the case for data collected in the sagittal plane. There was a tendency for the contralateral (unaffected) knee of ACLR participants to show a higher mean knee flexion moment in all activities compared to ACLR affected knees and control knees (Figure 3). The difference was not found to be statistically significant.

For peer review only

Discussion

ACL injury is often accompanied by other knee injuries.(23) Prevalence of associated meniscal damage and chondral lesions at the time of ACL injury can be as high as 65% and 23% respectively.(24) Associated knee injuries are thought to increase the incidence of OA from 0 – 13% in isolated ACL injury to 21 – 48%.(23) This may be because key knee structures such as menisci prevent cartilage wear by distributing loads and functioning as a shock absorber.(13) Our results suggest that high knee adduction moments may be a relevant risk factor in OA incidence in ACLR subjects only when associated knee injuries are present.

Three other studies have looked at peak knee adduction moment in ACLR and matched control subjects. Butler et al. found the ACLR group to have a higher peak knee adduction moment to controls.(15) This was also seen by our ACLR + group. Whereas the studies that followed, Webster et al. and Zabala et al. found the ACLR group to have

For peer review only - http://bmjopen.bmj.com/site/about/guidelines.xhtml

a reduced peak knee adduction moment to controls.(16, 17) This was seen by our ACLR - group. This disparity in studies may be a consequence of different exclusion criteria for ACLR subjects. Butler et al. did not exclude ACLR subjects with other knee injuries,(15) while the other two studies excluded subjects with ligament damage(16, 17) and also those with >25% of menisci loss.(17) We suggest that associated knee injuries are related to increased knee adduction moments in ACLR participants.

We found the difference between peak KAM in ACLR + and ACLR – to be statistically significant only during normal gait. We expected the difference to be higher during sloped walking, as it is more challenging than level walking. Change in terrain, muscle weakness, gait deficit and balance deficit are primary risk factors for falling.(25, 26) This suggests that ACLR participants need to adopt a conservative gait strategy while walking on a sloped surface to ensure safety. During challenging tasks such as downhill walking healthy participants increased their metabolic activity and implemented a conservative gait strategy to reduce the risk of falling.(25) This principle may also be applied by ACLR participants to ensure safety.

Both uphill and downhill walking require greater use of quadriceps muscle than on a level walkway.(26, 27) However in our study no statistically significant differences were observed between ACLR, ACLR +, ACLR – and control group in peak knee flexion and extension moments.

Two years after an ACLR surgery, differences in quadriceps strength between limbs are no longer seen.(25) All of the ACLR subjects had undergone reconstruction at least 1 year before taking part in this study, with an average of 4.5 years. This indicates that all

For peer review only - http://bmjopen.bmj.com/site/about/guidelines.xhtml

subjects may have had sufficient time to restore their quadriceps strength. Even though sagittal instability is thought to increase joint loads and lead to joint failure,(13, 28) it may not play a significant role in OA induction and progression after reconstruction.

An unexpected finding was the tendency for the contralateral knees of ACLR participants to have higher peak knee flexion and lower peak knee extension moment compared to the ACLR and control knee in all activities. This may be an adaptation to reduce loading on their ACLR knee. Patients with advanced knee OA also display this adaptation to reduce loading on their injured leg.(29) Even though this may present as a mechanism to slow the progression of OA, there are harmful implications associated with the contralateral leg. Weight bearing asymmetry may induce OA in the contralateral leg;(30) 37% (24/65 female patients) showed signs of radiographic OA in their contralateral leg, 12 years after ACL reconstruction.(31) This suggests that in this population unilateral injury changes joint function bilaterally.

In addition to our primary investigations we also investigated differences in KOOS and walking speed. There was no significant difference in Knee Osteoarthritis Outcome Score or gait speed between ACLR + and ACLR –. This indicates that high peak knee adduction moment in the injured leg does not affect our subjects' pain outcome, symptoms, activities of daily life, sport and recreation, knee related quality of life and gait speed. Thereby patient reported outcome measures and gait speed might not provide the clinician with any information about different gait adaptations.

Mundermann et al (2004) found a 10.2% reduction in maximum knee adduction moment when people with less severe OA reduced their walking speed from 1.2 meters/second to 0.8 meters per second.(32) However in this study the difference in peak knee

For peer review only - http://bmjopen.bmj.com/site/about/guidelines.xhtml

moments between normal (1.17 m/s) and slow gait speed (0.76 m/s) were not statistically significant. This may be due to our small sample size.

It is important to note that different knee injuries, rehabilitation protocols and time between injury and reconstruction are all thought to influence joint moments.(17) These are limitations that should be considered when examining the results presented here. In our study the difference in age between ACLR and control group was statistically significant. A multiple linear regression was carried out to show that age (p = 0.65) did not have a significant effect on ACLR knee adduction moment. Another limitation was our small sample size, in particular after dividing our ACLR group into ACLR + and ACLR – groups.

The ramp was set at an incline of 10 degrees because the transition from a level to slope walking strategy is thought to be around 5.5 degrees(27) and after the incline of 10 degrees no kinematic differences are seen in healthy participants.(27)

With regards to Vicon Motion capture system different skin marker placement and skin motion artifacts are thought to increase error.(6) We tried to reduce the effects of different skin marker placement by having only one researcher place all markers on each individual. In addition we used a model that used clusters to reduce the effects of skin motion artefacts.(22)

Conclusion

In conclusion, this study looked at peak moments in the sagittal and frontal plane during level and sloped walking for ACLR participants (subdivided into ACLR + and ACLR –)

For peer review only - http://bmjopen.bmj.com/site/about/guidelines.xhtml

For peer review only

and their contralateral legs. We found individuals who have other knee injuries associated with their ACLR knee exhibit higher peak adduction moments. This suggests that injuries to other key knee structures may play a bigger part in inducing OA than ACL injury alone. Contralateral knees appear to be functioning in such a way to reduce high moments in the ACLR knees, which may be relevant in the risk of OA development in both knees. These findings warrant a longitudinal study comparing the knee adduction moment between isolated ACLR injury and ACLR with additional knee injuries and the prevalence of premature OA.

Data sharing - no additional data available

Acknowledgements - The authors acknowledge support from Mr. R. Weinert-Aplin for the facilitation of the instrumented inclined walkway and conversion of the output data.

Competing interests - None

Funding - The authors are also thankful for the funding provided by the Wellcome Trust and the EPSRC.

Contributorship Statement - All authors helped with the study design. Raghav K Varma carried out data collection, statistical analysis and analysis of the data produced. He also drafted and revised the paper. He is guarantor. Lynsey D Duffell attained ethical approval, determined the methodology, helped with data analysis and drafted and revised the paper. Dinesh Nathwani helped with data collection and drafted and revised the paper. Alison H McGregor monitored the data collection for the whole trial, provided the data collection tools and drafted and revised the paper. Robert Weinert-Aplin designed the data collection tool.

For peer review only - http://bmjopen.bmj.com/site/about/guidelines.xhtml

References

1. Lyman S, Koulouvaris P, Sherman S et al. Epidemiology of anterior cruciate ligament reconstruction: trends, readmissions, and subsequent knee surgery. *J Bone Joint Surg Am* 2009;91:2321-8.
2. Matsumoto H, Suda Y, Otani T et al. Roles of the anterior cruciate ligament and the medial collateral ligament in preventing valgus instability. *J Orthop Sci* 2001;6:28-32.
3. Lohmander LS, Englund PM, Dahl LL et al. The long-term consequence of anterior cruciate ligament and meniscus injuries: osteoarthritis. *Am J Sports Med* 2007;35:1756-69.
4. Porat A, Roos EM, Roos H. High prevalence of osteoarthritis 14 years after an anterior cruciate ligament tear in male soccer players: a study of radiographic and patient relevant outcomes. *Ann Rheum Dis* 2004;63:269-73.
5. Andriacchi TP, Mündermann A. The role of ambulatory mechanics in the initiation and progression of knee osteoarthritis. *Curr Opin Rheumatol* 2006;18:514-8.
6. Stergiou N, Ristanis S, Moraiti C et al. Tibial rotation in anterior cruciate ligament (ACL)-deficient and ACL-reconstructed knees: a theoretical proposition for the development of osteoarthritis. *Sports Med* 2007;37:601-13.
7. Andriacchi TP, Mündermann A, Smith RL et al. A framework for the in vivo pathomechanics of osteoarthritis at the knee. *Ann Biomed Eng* 2004;32:447-57.
8. Hart JM, Ko JW, Konold T et al. Sagittal plane knee joint moments following anterior cruciate ligament injury and reconstruction: a systematic review. *Clin Biomech* 2010;25:277-83.

For peer review only

For peer review only - http://bmjopen.bmj.com/site/about/guidelines.xhtml

9. Georgoulis AD, Papadonikolakis A, Papageorgiou CD et al. Three-dimensional tibiofemoral kinematics of the anterior cruciate ligament-deficient and reconstructed knee during walking. *Am J Sports Med* 2003;31:75-9.

10. Noyes FR, Schipplein OD, Andriacchi TP et al. The anterior cruciate ligament-deficient knee with varus alignment. An analysis of gait adaptations and dynamic joint loadings. *Am J Sports Med* 1992;20:707-16.

11. Berchuck M, Andriacchi TP, Bach BR et al. Gait adaptations by patients who have a deficient anterior cruciate ligament. *J Bone Joint Surg Am* 1990;72:871-7.

12. Urbach D, Nebelung W, Becker R et al. Effects of reconstruction of the anterior cruciate ligament on voluntary activation of quadriceps femoris a prospective twitch interpolation study. *J Bone Joint Surg Br* 2001;83:1104-10.

13. Roos EM. Joint injury causes knee osteoarthritis in young adults. *Curr Opin Rheumatol* 2005;17:195-200.

14. Papageorgiou CD, Gil JE, Kanamori A et al. The biomechanical interdependence between the anterior cruciate ligament replacement graft and the medial meniscus. *Am J Sports Med* 2001;29:226-31.

15. Butler RJ, Minick KI, Ferber R et al. Gait mechanics after ACL reconstruction: implications for the early onset of knee osteoarthritis. *Br J Sports Med* 2009;43:366-70.

16. Webster KE, Feller JA. The knee adduction moment in hamstring and patellar tendon anterior cruciate ligament reconstructed knees. *Knee Surg Sports Traumatol Arthrosc* 2012;20:2214-9.

For peer review only

17. Zabala ME, Favre J, Scanlan SF et al. Three-dimensional knee moments of ACL reconstructed and control subjects during gait, stair ascent, and stair descent. *J Biomech* 2013;46:515-20.

18. Miyazaki T, Wada M, Kawahara H et al. Dynamic load at baseline can predict radiographic disease progression in medial compartment knee osteoarthritis. *Ann Rheum Dis* 2002;61:617-22.

19. Roos EM, Roos HP, Lohmander LS et al. Knee Injury and Osteoarthritis Outcome Score (KOOS)— development of a self-administered outcome measure. *J Orthop Sports Phys Ther* 1998;28:88–96.

20. Cleather DJ, Bull AM Influence of inverse dynamics methods on the calculation of inter-segmental moments in vertical jumping and weightlifting. *Biomed Eng Online* 2010;9:74

21. Cleather DI, Bull AM Lower-extremity musculoskeletal geometry affects the calculation of patellofemoral forces in vertical jumping and weightlifting. *Proc Inst Mech Eng H* 2010;224:1073-1083

22. Hope, N., Duffell, L. D., and McGregor, A. H. Validation of a new model to calculate joint kinematics during gait. *European Society of Biomechanics Conference Proceedings* 1-7-2012.

23. Claes S, Hermie L, Verdonk R et al. Is osteoarthritis an inevitable consequence of anterior cruciate ligament reconstruction? A meta- analysis. *Knee Surg Sports Traumatol Arthrosc* 2013;21:1967-76.

24. Louboutin H, Debarge R, Richou J et al. Osteoarthritis in patients with anterior cruciate ligament rupture: a review of risk factors. *Knee* 2009;16:239-44.

For peer review only - http://bmjopen.bmj.com/site/about/guidelines.xhtml

25. Monsch ED, Franz CO, Dean JC. The effects of gait strategy on metabolic rate and indicators of stability during downhill walking. *J Biomech* 2012;45:1928-33.
26. Mizner RL, Snyder-Mackler L. Altered loading during walking and sit-to-stand is affected by quadriceps weakness after total knee arthroplasty. *J Orthop Res* 2005;23:1083-90.
27. A.N. Lay, C.J. Hass, R.J. Gregor. The effects of sloped surfaces on locomotion: a kinematic and kinetic analysis. *Journal of Biomechanics* 2006;39:1621–1628
28. Gottschall JS, Nichols TR. Neuromuscular strategies for the transitions between level and hill surfaces during walking. *Philos Trans R Soc Lond B Biol Sci* 2011;366:1565-79.
29. Creaby MW, Bennell KL, Hunt MA. Gait differs between unilateral and bilateral knee osteoarthritis. *Arch Phys Med Rehabil* 2012;93(5):822-7.
30. Christiansen CL, Stevens-Lapsley JE. Weight-bearing asymmetry in relation to 378 measures of impairment and functional mobility for people with knee osteoarthritis. *Arch Phys Med Rehabil* 2010;91:1524-8.
31. Lohmander, L.S., Ostenberg, A., Englund, M. et al. High prevalence of knee osteoarthritis, pain, and functional limitations in female soccer players twelve years after anterior cruciate ligament injury. *Arthritis and Rheumatology* 2004;50:3145–3152.
32. Mündermann A, Dyrby CO, Hurwitz DE et al. Potential strategies to reduce medial compartment loading in patients with knee osteoarthritis of varying severity: reduced walking speed. *Arthritis Rheum* 2004;50:1172-8.

For peer review only - http://bmjopen.bmj.com/site/about/guidelines.xhtml

Figures

Fig. 1 Steel framed ramp covered in plywood, set an incline of 10 degrees.

Fig. 2 Peak adduction moments in **a)** level walking and **b)** slope walking for ACLR, ACLR +, ACLR – and control group. Asterisk indicates significance ($p = 0.042$).

Fig. 3, a) Peak flexion moment in all activities for ACLR, contralateral and control group. § represents decline gait to be significantly higher than all other activities ($p<0.01$). Asterisk indicates significance, $p<0.05$ **b)** Peak extension moment in all activities for ACLR, contralateral and control group. ~ represents decline gait to be significantly lower than normal and incline gait, $p<0.05$

For peer review only

For peer review only - http://bmjopen.bmj.com/site/about/guidelines.xhtml

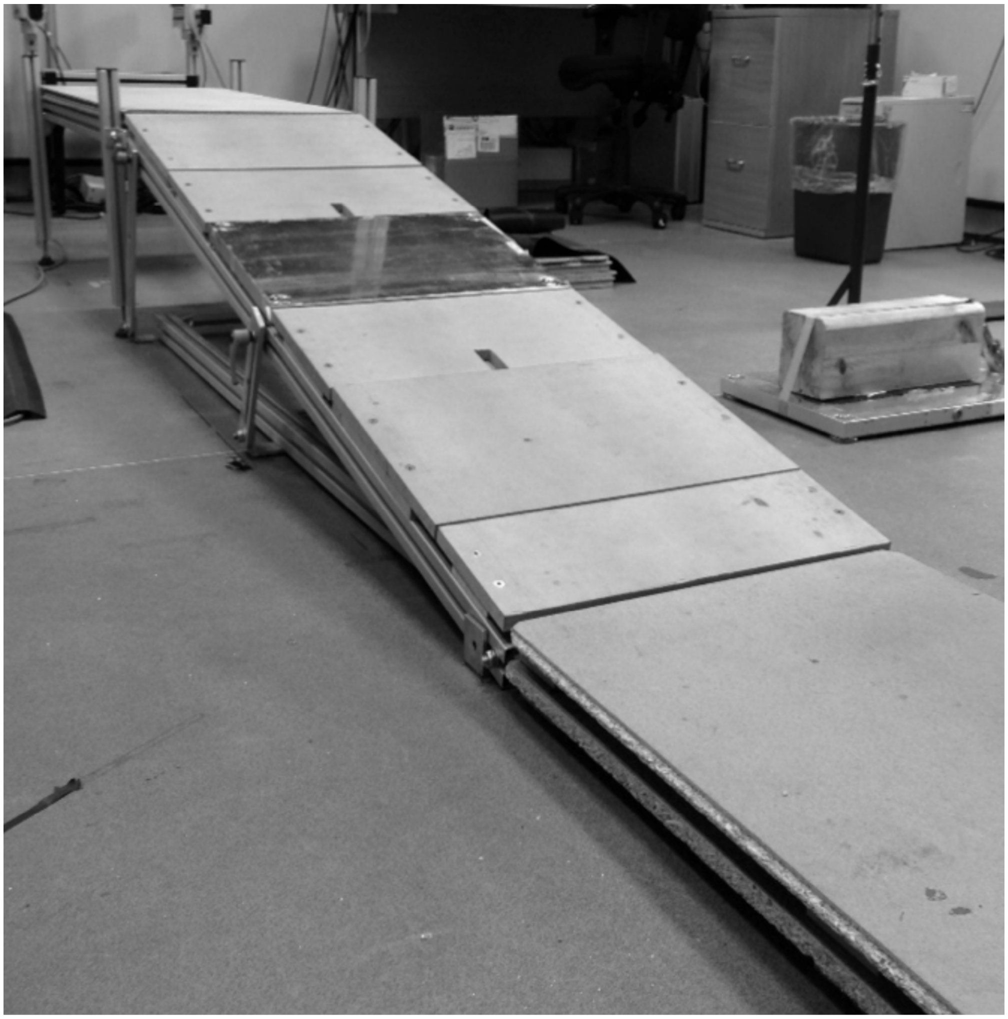

Steel framed ramp covered in plywood, set an incline of 10 degrees.
188x190mm (300 x 300 DPI)

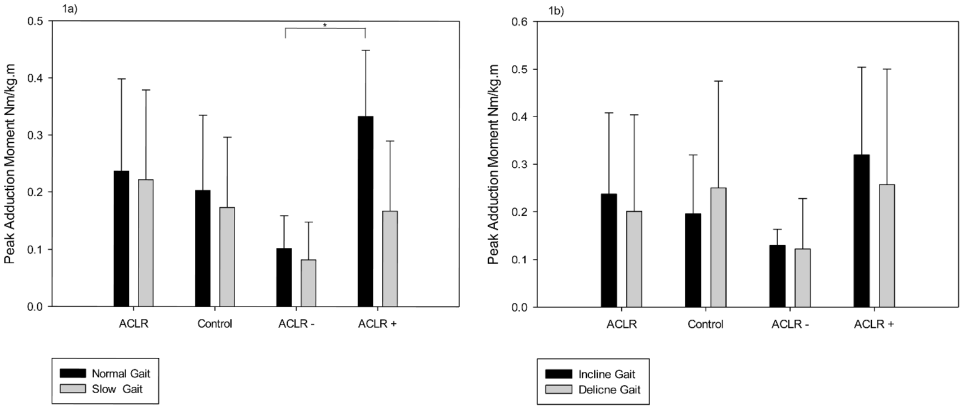

Peak adduction moments in a) level walking and b) slope walking for ACLR, ACLR +, ACLR – and control group. Asterisk indicates significance (p = 0.042).
533x254mm (300 x 300 DPI)

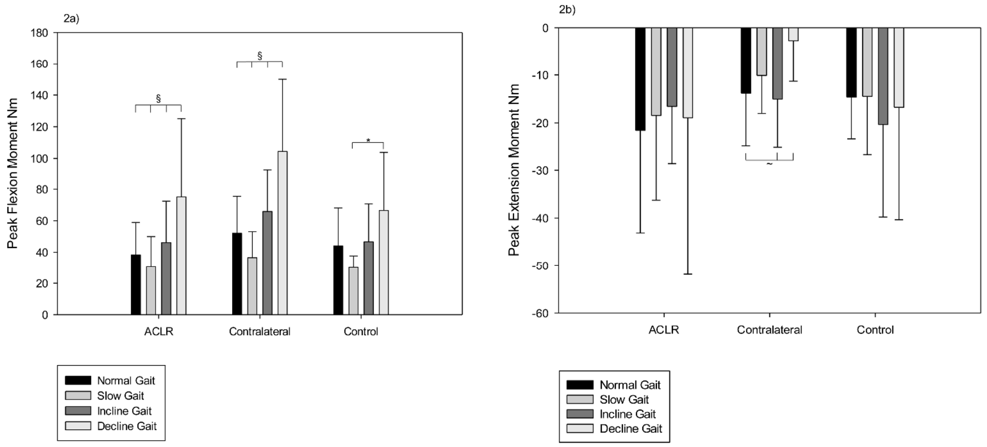

a) Peak flexion moment in all activities for ACLR, contralateral and control group. § represents decline gait to be significantly higher than all other activities ($p<0.01$). Asterisk indicates significance, $p<0.05$ b) Peak extension moment in all activities for ACLR, contralateral and control group. ~ represents decline gait to be significantly lower than normal and incline gait, $p<0.05$
508x254mm (300 x 300 DPI)

STROBE Statement—checklist of items that should be included in reports of observational studies

	Item No	Recommendation
**Title and abstract**	1	(*a*) Indicate the study's design with a commonly used term in the title or the abstract
		(*b*) Provide in the abstract an informative and balanced summary of what was done and what was found
**Introduction**		
Background/rationale	2	Explain the scientific background and rationale for the investigation being reported
Objectives	3	State specific objectives, including any prespecified hypotheses
**Methods**		
Study design	4	Present key elements of study design early in the paper
Setting	5	Describe the setting, locations, and relevant dates, including periods of recruitment, exposure, follow-up, and data collection
Participants	6	(*a*) *Cohort study*—Give the eligibility criteria, and the sources and methods of selection of participants. Describe methods of follow-up<br>*Case-control study*—Give the eligibility criteria, and the sources and methods of case ascertainment and control selection. Give the rationale for the choice of cases and controls<br>*Cross-sectional study*—Give the eligibility criteria, and the sources and methods of selection of participants
		(*b*) *Cohort study*—For matched studies, give matching criteria and number of exposed and unexposed<br>*Case-control study*—For matched studies, give matching criteria and the number of controls per case
Variables	7	Clearly define all outcomes, exposures, predictors, potential confounders, and effect modifiers. Give diagnostic criteria, if applicable
Data sources/ measurement	8*	For each variable of interest, give sources of data and details of methods of assessment (measurement). Describe comparability of assessment methods if there is more than one group
Bias	9	Describe any efforts to address potential sources of bias
Study size	10	Explain how the study size was arrived at
Quantitative variables	11	Explain how quantitative variables were handled in the analyses. If applicable, describe which groupings were chosen and why
Statistical methods	12	(*a*) Describe all statistical methods, including those used to control for confounding
		(*b*) Describe any methods used to examine subgroups and interactions
		(*c*) Explain how missing data were addressed
		(*d*) *Cohort study*—If applicable, explain how loss to follow-up was addressed<br>*Case-control study*—If applicable, explain how matching of cases and controls was addressed<br>*Cross-sectional study*—If applicable, describe analytical methods taking account of sampling strategy
		(*e*) Describe any sensitivity analyses

Continued on next page

For peer review only

**Results**		
Participants	13*	(a) Report numbers of individuals at each stage of study—eg numbers potentially eligible, examined for eligibility, confirmed eligible, included in the study, completing follow-up, and analysed
		(b) Give reasons for non-participation at each stage
		(c) Consider use of a flow diagram
Descriptive data	14*	(a) Give characteristics of study participants (eg demographic, clinical, social) and information on exposures and potential confounders
		(b) Indicate number of participants with missing data for each variable of interest
		(c) *Cohort study*—Summarise follow-up time (eg, average and total amount)
Outcome data	15*	*Cohort study*—Report numbers of outcome events or summary measures over time
		Case-control study—Report numbers in each exposure category, or summary measures of exposure
		Cross-sectional study—Report numbers of outcome events or summary measures
Main results	16	(*a*) Give unadjusted estimates and, if applicable, confounder-adjusted estimates and their precision (eg, 95% confidence interval). Make clear which confounders were adjusted for and why they were included
		(*b*) Report category boundaries when continuous variables were categorized
		(*c*) If relevant, consider translating estimates of relative risk into absolute risk for a meaningful time period
Other analyses	17	Report other analyses done—eg analyses of subgroups and interactions, and sensitivity analyses
**Discussion**		
Key results	18	Summarise key results with reference to study objectives
Limitations	19	Discuss limitations of the study, taking into account sources of potential bias or imprecision. Discuss both direction and magnitude of any potential bias
Interpretation	20	Give a cautious overall interpretation of results considering objectives, limitations, multiplicity of analyses, results from similar studies, and other relevant evidence
Generalisability	21	Discuss the generalisability (external validity) of the study results
**Other information**		
Funding	22	Give the source of funding and the role of the funders for the present study and, if applicable, for the original study on which the present article is based

*Give information separately for cases and controls in case-control studies and, if applicable, for exposed and unexposed groups in cohort and cross-sectional studies.

Note: An Explanation and Elaboration article discusses each checklist item and gives methodological background and published examples of transparent reporting. The STROBE checklist is best used in conjunction with this article (freely available on the Web sites of PLoS Medicine at http://www.plosmedicine.org/, Annals of Internal Medicine at http://www.annals.org/, and Epidemiology at http://www.epidem.com/). Information on the STROBE Initiative is available at www.strobe-statement.org.

For peer review only

For peer review only - http://bmjopen.bmj.com/site/about/guidelines.xhtml

BMJ Open

Knee moments of anterior cruciate ligament reconstructed and control participants during normal and inclined walking.

Journal:	*BMJ Open*
Manuscript ID:	bmjopen-2013-004753.R1
Article Type:	Research
Date Submitted by the Author:	25-Apr-2014
Complete List of Authors:	Varma, Raghav; Imperial College London, MSK Lab<br>Duffell, Lynsey; Imperial College London, MSK Lab<br>Nathwani, Dinesh; Imperial College London, MSK Lab<br>McGregor, Alison; Imperial College London, MSK Lab
**Primary Subject Heading**:	Sports and exercise medicine
Secondary Subject Heading:	Rehabilitation medicine, Sports and exercise medicine
Keywords:	Knee < ORTHOPAEDIC & TRAUMA SURGERY, Orthopaedic sports trauma < ORTHOPAEDIC & TRAUMA SURGERY, SPORTS MEDICINE

SCHOLARONE™
Manuscripts

For peer review only

Title: Knee moments of anterior cruciate ligament reconstructed and control participants during normal and inclined walking.

Authors: Raghav K Varma[1], Lynsey D Duffell[1], Dinesh Nathwani[1] & Alison H McGregor [1].

Affiliations: 1) MSK Lab, Imperial College London, UK

Corresponding Author:

Professor Alison McGregor

MSK Lab

Imperial College London

Floor 7, Laboratory Block

Charing Cross Hospital, Fulham Palace Road

London, W6 8RP

T: 020 3313 8834

F: 020 3313 8835

E: a.mcgregor@imperial.ac.uk

Word count: 3056

Key words: Anterior cruciate ligament reconstruction, inclined walkway, adduction moment and gait analysis.

For peer review only

Abstract

Objectives

Prior injury to the knee, particularly anterior cruciate ligament (ACL) injury is known to predispose to premature osteoarthritis (OA). The study sought to explore if there was a biomechanical rationale for this process by investigating changes in external knee moments between people with a history of ACL injury and uninjured subjects during walking; i) on different surface inclines and; ii) at different speeds. In addition we assessed functional differences between the groups.

Participants

Twelve subjects who had undergone ACL reconstruction (ACLR) and 12 volunteers with no history of knee trauma or injury were recruited into this study. Peak knee flexion and adduction moments were assessed during flat (normal and slow speed), uphill and downhill walking using a inclined walkway with an embedded Kistler Force plate, and a ten camera Vicon motion capture system. Knee osteoarthritis outcome score (KOOS) was used to assess function. MANOVA was used to examine statistical differences in gait and KOOS outcomes.

Results

No significant difference was observed in the peak knee adduction moment between ACLR and control participants however, in further analysis, MANOVA revealed that ACLR participants with an additional meniscal tear or collateral ligament damage (7 subjects) had a significantly higher adduction moment (0.33 ± 0.12 Nm/kg.m) when compared to those with isolated ACLR (5 subjects, 0.1 ± 0.057 Nm/kg.m) during gait at

For peer review only - http://bmjopen.bmj.com/site/about/guidelines.xhtml

their normal speed ($p<0.05$). A similar (non-significant) trend was seen during slow, uphill and downhill gait.

Conclusion

Subjects with an isolated ACLR had a reduced adductor moment rather an increased moment, thus questioning prior theories on OA development. In contrast those subjects who had sustained associated trauma to other key knee structures were observed to have an increased adduction moment. Additional injury concurrent with an ACL rupture may lead to a higher predisposition to osteoarthritis than isolated ACL deficiency alone.

Article summary

Strengths and limitations of this study

- To our knowledge this is the first report looking at external moments during inclined and declined walking for ACL reconstruction participants.
- In addition to looking into the external moments of the affected and unaffected knee, this study also looked at the effect of gait speed on external moments and differences in knee osteoarthritis outcome score (KOOS) for each group.
- This study provides a potential explanation for the disparity seen in previous studies looking into peak knee adduction moment in ACLR and matched control subjects.
- This study suggests that injuries to other key knee structures may play a bigger part in inducing osteoarthritis than ACL injury alone.

For peer review only

For peer review only - http://bmjopen.bmj.com/site/about/guidelines.xhtml

- One limitation to this study is the small sample size, in particular after dividing our ACLR group into ACLR + and ACLR – groups.

Introduction

Anterior Cruciate Ligament (ACL) injuries are common, exceeding 100,000 annual cases in the United States.(1) The majority are sports related injuries, and lead to knee instability as a result of increased anterior tibial translation and anterolateral rotation.(2) ACL reconstruction (ACLR) is the primary treatment for an ACL rupture and permits return to a range of high-level activities including sport. It is accepted that people with ACL injuries, including those who undergo surgical reconstruction, are prone to further knee degeneration(3) and early OA.(4,5) Lohmander et al. (2007) reviewed 127 publications and determined that the overall mean incidence of developing OA after an ACL injury with/without reconstruction to be over 50%(3) with the majority noting radiographic signs of OA 10 years after injury.(3) Gait biomechanics are considered to play a vital part in knee joint degeneration, (5, 6) with altered kinematics and kinetics changing the distribution of mechanical load on the knee. (7) This in turn is postulated to lead to cartilage wear(5 - 7) and eventually knee osteoarthritis.

There is consensus amongst researchers that ACL deficient patients employ different gait strategies.(8) In vivo studies have found reduced knee flexion,(5) increased internal tibial rotation(5, 9) and increased knee adduction moment(10) during level walking, to be the three main changes in external knee moments following an ACL rupture. Furthermore, research has indicated that ACL reconstruction does not restore normal knee mechanics(11). Berchuck et al. in 1990(12) noted reduced knee flexion during

For peer review only - http://bmjopen.bmj.com/site/about/guidelines.xhtml

normal gait, indicating a coping strategy termed quadriceps avoidance gait. Anterior displacement of tibia through the contraction of quadriceps is balanced by the ACL when the knee is at an angle of 0 to 45 degrees.(12) People with ACL rupture and/or reconstruction are found to have quadriceps activation deficits(13), which may be due to a central regulatory mechanism to avoid further joint damage by these muscle groups. Gait adaptations in the sagittal plane can lead to knee joint instability and ligament laxity.(14) This may result in osteoarthritis initiation and progression.(14)

High moments in frontal and transverse planes of the knee have been linked to OA.(5) ACLR has been shown to restore rotational stability,(9, 15) however high knee adduction moments (KAM) after reconstruction have been observed(16) but such changes are not universally agreed.(17, 18) This is of particular importance since a 1% increase in adduction moment at the knee is thought to increase the risk of knee OA by 6.5 times.(19) The discrepancies in previous studies may be due to different walking speeds, and higher KAM may only be evident during more challenging tasks. Hence in this study we aimed to gain a better understanding of peak knee moments in the frontal and sagittal plane during gait at different speeds and inclines. Our primary aim is to compare peak knee moments in the sagittal and frontal plane of ACLR participants to healthy controls on sloped surfaces, with a view to exploring the biomechanical basis for the observation that ACL injury predisposes to OA. Our secondary aim was to investigate the effect of speed on peak moments. Finally, we compared functional outcome scores between groups using the Knee injury and Osteoarthritis Outcome Score (KOOS).

For peer review only

Methods

This cross-sectional study explored peak knee moments between ACLR and healthy control subjects during slope walking. The study was approved by Imperial College Research Ethics Committee. We used the STROBE statement as a checklist for our observational study. (20)

A total number of 24 subjects participated in this study and written informed consent obtained, details are provided in Table 1. The ACLR inclusion criteria were; aged between 18 – 60 years, Body mass index (BMI) < 30kg/m^2, a complete, unilateral ACL rupture followed by a single bundle hamstring auto graft reconstruction that was performed at least one-year ago with no history of knee trauma or injury to their contralateral leg. Subjects who were unable to walk comfortably on a 10-degree incline walkway were also excluded. The control group did not have any muscular or neurological lower limb pathology and were matched to the ACLR subjects with respect to gender, activity, height, weight and their dominant leg (leg preference for kicking). All subject completed the Knee injury and Osteoarthritis Outcome Score (KOOS)(21). We measured the subject's activity levels using Tegner Activity scale (22).

A three-dimensional motion analysis system (Vicon MX™ T-20 System, Vicon, Oxford) was used to collect kinematic data for normal, slow, upslope and downslope gait. This software used 10 motion capture cameras to pick up 35 reflective markers at a sampling rate of 100Hz. The reflective markers were placed bilaterally on the head of 2nd metatarsal, head of 5th metatarsal, head of talus, calcaneal tuberosity, medial and lateral malleolus, medial and lateral femoral epicondyle, anterior superior iliac spine, posterior superior iliac spine, acromion and one single marker on the manubrium. Marker clusters

For peer review only - http://bmjopen.bmj.com/site/about/guidelines.xhtml

(3 reflective markers on each) were affixed bilaterally to the calf and thigh. Kinetic data (ground reaction force) were collected using portable force plates (Kistler Instruments AG, Winterthur, Switzerland) at a sampling rate of 1000 Hz and was synchronised with the camera data.

The subjects were asked to walk barefoot. A 5 minute self-directed warm up allowed the subjects to familiarise themselves with each task.

A 7-meter long walkway was used, 2.5-meters of which could be raised to form a ramp, at an incline of 10 degrees. It was constructed from a steel frame and covered with plywood, with one portable force plate embedded in the centre (Figure 1). Participants were asked to walk at a self-selected pace both uphill and downhill. The ramp was then removed to create a level walkway. All the participants were asked to walk at a self-selected pace and at a pace, which they consider to be slow. Each task was repeated until both feet made complete contact with the middle of the force plate at least three times.

All data was time normalised to one gait cycle. The data was analysed from the stance phase of the gait cycle; when the ground reaction force reached more than 40N (heel strike) to when it dropped to less than 40N (toe off). A fourth order Butterworth Filter at a cut off (12 Hz) was used to reduce noise. Joint angles and moments were calculated from the position of the reflective markers and the ground reaction force data using a custom model written in body builder software.(23-25) Peak moments in the sagittal and frontal planes of the knee were extracted using MATLAB (R2013b) software.

For peer review only

Unpaired Student's t-tests were used to determine any significant differences in demographics between ACLR and control group. MANOVA test was used to calculate significant differences in all other parameters. Tukey HSD approach was used to establish significance, with the alpha value set at 0.05. All statistics were carried out using SPSS version 22.

Results

12 ACLR participants (9 male and 3 female) and 12 control (9 male and 3 female) were recruited for this study. There were no significant differences between groups in age, height, weight and Tegner Activity Scale (Table 1). The mean time since reconstruction surgery was 4 years and 6 months.

Table 1 Subject characteristics, activity level and time since surgery. 12 participants in ACLR group and 12 in control group

	ACLR (SD)	Control (SD)	Unpaired T-test
Age (yr)	30.5 (8.68)	24.8 (8.81)	P = 0.125
Height (m)	1.76 (0.13)	1.73 (0.11)	P = 0.547
Weight (kg)	75 (11.13)	71.6 (11.2)	P = 0.464
Tegner Activity Scale	6.25 (1.82)	6.08 (1.93)	P = 0.826
Time since surgery (yr)	4.5 (3.5)	NA	

ACLR, Anterior cruciate ligament reconstruction

We further divided our ACLR group into two; subjects that had additional cartilage, meniscus or ligament damage in their ACLR leg (ACLR + group; 7 subjects) and subjects with isolated ACL injuries (ACLR – group; 5 subjects). The additional knee injuries to the ACL rupture are meniscal tear (3 participants), cartilage damage (3 participants) and torn MCL (1 participant).

For peer review only

No statistically significant differences were found in peak knee adduction moment between ACLR and control participants during uphill and downhill gait, and during gait at normal and slow walking speeds on a flat surface (Figure 2). Further analysis revealed that ACLR participants with meniscal tear, cartilage damage or medial collateral ligament damage (ACLR +) had significantly higher knee adduction moment (0.33 ± 0.12 Nm/kg.m) during gait on a flat surface at a normal walking speed compared to those participants with an isolated ACL injury (ACLR -, 0.1 ± 0.057 Nm/kg.m), p = 0.042 (Figure 2).

This was not the case for data collected in the sagittal plane. There was a tendency for the contralateral (unaffected) knee of ACLR participants to show a higher mean knee flexion moment in all activities compared to ACLR affected knees and control knees (Figure 3). The difference was not found to be statistically significant.

Table 2 shows that there were no significant differences in gait speed in normal or slow walking between any groups. The control group had significantly higher scores in all of the KOOS domains apart from activities of daily life compared to the ACLR group (Table 3). No significant KOOS differences were seen between ACLR + and ACLR – groups.

Table 2 Gait speed during normal and slow, level walking tasks.

	ACLR	Control	ACLR +	ACLR -	P = value
Gait normal speed	1.17 (0.13)	1.20 (0.11)	1.18 (0.15)	1.16 (0.11)	P = 0.940
Gait slow speed	0.76 (0.13)	0.75 (0.11)	0.78 (0.16)	0.74 (0.09)	P = 0.885

ACLR, Anterior cruciate ligament reconstruction; ACLR +, subjects with other knee injuries in their ACLR leg; ACLR -, subjects with isolated ACL injuries.
Data are mean (SD).

For peer review only - http://bmjopen.bmj.com/site/about/guidelines.xhtml

Table 3 Knee osteoarthritis outcome score (KOOS) with standard deviation (SD) for each domain recorded for each group.

KOOS Outcome	ACLR (SD) (n=12)	Control (SD) (n=12)	ACLR + (SD) (n=7)	ACLR - (SD) (n=5)	Significant values
Pain	88.4 (9.32)	99.1 (3.2)	87.5 (8.83)	89.4 (10.8)	Control vs ACLR: P = 0.010 Control vs ACLR(+): P = 0.019
Symptoms	83.1 (11.4)	98.2 (3.19)	85.1 (12.7)	80.7 (10.6)	Control vs all other groups: P < 0.05
Activities of daily life	96.3 (5.63)	100 (0)	98 (3)	94.4 (7.7)	No significant differences
Sport and recreation	83.8 (16.9)	99.6 (1.4)	89.1 (7.4)	77.4 (23)	Control vs ACLR: P = 0.006 Control vs ACLR(-): P = .003
Knee related QOL	64.5 (23.2)	100 (0)	64.6 (23.3)	70 (26.7)	Control vs all other groups: P < 0.05

ACLR, Anterior cruciate ligament reconstruction; ACLR +, subjects with other knee injuries in their ACLR leg; ACLR -, subjects with isolated ACL injuries.
Data are mean (SD).

Discussion

In the frontal plane we found no statistically significant differences between our ACLR participants and controls. However ACLR subjects who had sustained associated trauma to other key knee structures (meniscal, collateral ligament and chondral damage) were observed to have a higher adduction moment during gait on a flat surface at normal walking speed when compared to subjects with isolated ACLR. In the sagittal

For peer review only - http://bmjopen.bmj.com/site/about/guidelines.xhtml

plane there was a tendency for ACLR participants to have higher peak knee flexion moment in their contralateral leg during all activities.

Previous studies that have investigated peak knee adduction moment in ACLR and matched control subjects have provided mixed results.(16,17,18) We investigated similar and more challenging gait set-ups (by altering incline), as well as the effects of differences in walking speed, in order to explore the biomechanical basis for the observation that ACL injury predisposes to OA. Our data showed no significant differences in KAM between our ACLR participants and control subjects under all conditions. This indicates that providing more challenging gait set-ups such as sloped walking, where a higher range of motion in the sagittal plane is required, does not emphasize differences between ACLR and control subjects. Based on our findings, the discrepancies in previous studies appear not to be related to the difficulty of the task or differences in walking speed.

ACL injury is often accompanied by other knee injuries.(26) Prevalence of associated meniscal damage and chondral lesions at the time of ACL injury can be as high as 65% and 23% respectively.(27) Associated knee injuries are thought to increase the incidence of OA from 0 – 13% in isolated ACL injury to 21 – 48%.(28) This may be because key knee structures such as menisci prevent cartilage wear by distributing loads and functioning as a shock absorber.(14) Our results suggest that people with ACLR injuries with associated knee injuries experience higher knee adduction moments than people with isolated ACLR injuries.

For peer review only - http://bmjopen.bmj.com/site/about/guidelines.xhtml

Of the three previous studies that have looked at peak KAM in ACLR and matched control subjects, Butler et al. found the ACLR group to have a higher peak KAM compared to controls.(16) This was also seen by our ACLR + group. In the studies that followed, Webster et al. and Zabala et al. found the ACLR group to have a reduced peak knee adduction moment to controls.(17, 18) This was seen by our ACLR – group (Figure 2). This disparity in studies may therefore be a consequence of different exclusion criteria for ACLR subjects. Butler et al. did not exclude ACLR subjects with other knee injuries,(16) while the other two studies excluded subjects with ligament damage(17, 18) and also those with >25% of menisci loss.(18) We suggest that associated knee injuries are related to increased knee adduction moments in ACLR participants.

We found the difference between peak KAM in ACLR + and ACLR – to be statistically significant only during normal gait. We expected the difference to be higher during sloped walking, as it is more challenging than level walking. Change in terrain, muscle weakness, gait deficit and balance deficit are primary risk factors for falling.(29, 30) This suggests that ACLR participants need to adopt a conservative gait strategy while walking on a sloped surface to ensure safety. During challenging tasks such as downhill walking healthy participants increased their metabolic activity and implemented a conservative gait strategy to reduce the risk of falling.(29) This principle may also be applied by ACLR participants to ensure safety.

In the sagittal plane, no statistically significant differences were observed between ACLR, ACLR +, ACLR – and control group in peak knee flexion and extension moments. Both uphill and downhill walking require greater use of quadriceps muscle than on a level walkway.(30, 31) Two years after an ACLR surgery, differences in quadriceps

For peer review only - http://bmjopen.bmj.com/site/about/guidelines.xhtml

strength between limbs are no longer seen.(29) All of the ACLR subjects had undergone reconstruction at least 1 year before taking part in this study, with an average of 4.5 years. This indicates that all subjects may have had sufficient time to restore their quadriceps strength. Even though sagittal instability is thought to increase joint loads and lead to joint failure,(14, 32) it may not play a significant role in OA induction and progression after reconstruction.

An unexpected finding was the tendency for the contralateral knees of ACLR participants to have higher peak knee flexion and lower peak knee extension moment compared to the ACLR and control knee in all activities. This may be an adaptation to reduce loading on their ACLR knee. Patients with advanced knee OA also display this adaptation to reduce loading on their injured leg.(33) Even though this may present as a mechanism to slow the progression of OA, there are harmful implications associated with the contralateral leg. Weight bearing asymmetry may induce OA in the contralateral leg;(34) 37% (24/65 female patients) showed signs of radiographic OA in their contralateral leg, 12 years after ACL reconstruction.(35) This suggests that in this population unilateral injury changes joint function bilaterally.

In addition to our primary investigations we also investigated differences in KOOS and walking speed. There was no significant difference in KOOS or gait speed between ACLR + and ACLR –. This indicates that high peak knee adduction moment in the injured leg does not affect our subjects' pain outcome, symptoms, activities of daily life, sport and recreation, knee related quality of life and gait speed. Thereby patient reported outcome measures and gait speed might not provide the clinician with any information about different gait adaptations.

For peer review only - http://bmjopen.bmj.com/site/about/guidelines.xhtml
For peer review only

Mundermann et al (2004) found a 10.2% reduction in maximum knee adduction moment when people with less severe OA reduced their walking speed from 1.2 meters/second to 0.8 meters per second.(36) However in the current study the difference in peak knee moments between normal (1.17 m/s) and slow gait speed (0.76 m/s) were not statistically significant. This may be due to our small sample size.

It is important to note that different knee injuries, rehabilitation protocols and time between injury and reconstruction are all thought to influence joint moments.(18) These are limitations that should be considered when examining the results presented here. Additionally our participants were not recruited straight after their ACLR, some participants may have had further injury or pathological changes within the joint since the reconstruction. Another limitation was our small sample size, in particular after dividing our ACLR group into ACLR + and ACLR – groups.

The ramp was set at an incline of 10 degrees because the transition from a level to slope walking strategy is thought to be around 5.5 degrees(31) and after the incline of 10 degrees no kinematic differences are seen in healthy participants.(31)

With regards to Vicon Motion capture system different skin marker placement and skin motion artifacts are thought to increase error.(6) We tried to reduce the effects of different skin marker placement by having only one researcher place all markers on each individual. In addition we used a model that used clusters to reduce the effects of skin motion.(25)

For peer review only

For peer review only - http://bmjopen.bmj.com/site/about/guidelines.xhtml

Conclusion

In conclusion, this study found no significant differences in peak moments in the frontal and sagittal plane during level and sloped walking for ACLR compared with control participants. However, we noted that individuals who have other knee injuries associated with their ACLR knee exhibit higher peak adduction moments during level walking at their normal speed. This suggests that injuries to other key knee structures may play a bigger part in inducing OA than ACL injury alone, although this requires further investigation with a larger sample size. Our data also suggest that the contralateral knee appears to be functioning in such a way to reduce high moments in the ACLR knees, which may be relevant in the risk of OA development in both knees. These findings warrant a longitudinal study comparing the knee adduction moment between isolated ACLR injury and ACLR with additional knee injuries and the prevalence of premature OA.

For peer review only

For peer review only - http://bmjopen.bmj.com/site/about/guidelines.xhtml

Acknowledgements - The authors acknowledge support from Mr. R. Weinert-Aplin for the facilitation of the instrumented inclined walkway and conversion of the output data.

Funding - The authors are also thankful for the funding provided by the Wellcome Trust and the EPSRC.

Contributorship Statement - All authors helped with the study design. Raghav K Varma carried out data collection, statistical analysis and analysis of the data produced. He also drafted and revised the paper. He is guarantor. Lynsey D Duffell attained ethical approval, determined the methodology, helped with data analysis and drafted and revised the paper. Dinesh Nathwani helped with data collection and drafted and revised the paper. Alison H McGregor monitored the data collection for the whole trial, provided the data collection tools and drafted and revised the paper. Robert Weinert-Aplin designed the data collection tool.

Competing interests - None

Data sharing - No additional data available

For peer review only

References

1. Lyman S, Koulouvaris P, Sherman S et al. Epidemiology of anterior cruciate ligament reconstruction: trends, readmissions, and subsequent knee surgery. *J Bone Joint Surg Am* 2009;91:2321-8.
2. Matsumoto H, Suda Y, Otani T et al. Roles of the anterior cruciate ligament and the medial collateral ligament in preventing valgus instability. *J Orthop Sci* 2001;6:28-32.
3. Lohmander LS, Englund PM, Dahl LL et al. The long-term consequence of anterior cruciate ligament and meniscus injuries: osteoarthritis. *Am J Sports Med* 2007;35:1756-69.
4. Porat A, Roos EM, Roos H. High prevalence of osteoarthritis 14 years after an anterior cruciate ligament tear in male soccer players: a study of radiographic and patient relevant outcomes. *Ann Rheum Dis* 2004;63:269-73.
5. Andriacchi TP, Mündermann A. The role of ambulatory mechanics in the initiation and progression of knee osteoarthritis. *Curr Opin Rheumatol* 2006;18:514-8.
6. Stergiou N, Ristanis S, Moraiti C et al. Tibial rotation in anterior cruciate ligament (ACL)-deficient and ACL-reconstructed knees: a theoretical proposition for the development of osteoarthritis. *Sports Med* 2007;37:601-13.
7. Andriacchi TP, Mündermann A, Smith RL et al. A framework for the in vivo pathomechanics of osteoarthritis at the knee. *Ann Biomed Eng* 2004;32:447-57.
8. Hart JM, Ko JW, Konold T et al. Sagittal plane knee joint moments following anterior cruciate ligament injury and reconstruction: a systematic review. *Clin Biomech* 2010;25:277-83.

For peer review only

For peer review only - http://bmjopen.bmj.com/site/about/guidelines.xhtml

9. Georgoulis AD, Papadonikolakis A, Papageorgiou CD et al. Three-dimensional tibiofemoral kinematics of the anterior cruciate ligament-deficient and reconstructed knee during walking. *Am J Sports Med* 2003;31:75-9.
10. Noyes FR, Schipplein OD, Andriacchi TP et al. The anterior cruciate ligament-deficient knee with varus alignment. An analysis of gait adaptations and dynamic joint loadings. *Am J Sports Med* 1992;20:707-16.
11. Gokeler A, Benjaminse A, van Eck CF, et al. Return of normal gait as an outcome measurement in acl reconstructed patients. A systematic review. Int J Sports Phys Ther. 2013 Aug;8(4):441-51.
12. Berchuck M, Andriacchi TP, Bach BR et al. Gait adaptations by patients who have a deficient anterior cruciate ligament. *J Bone Joint Surg Am* 1990;72:871-7.
13. Urbach D, Nebelung W, Becker R et al. Effects of reconstruction of the anterior cruciate ligament on voluntary activation of quadriceps femoris a prospective twitch interpolation study. *J Bone Joint Surg Br* 2001;83:1104-10.
14. Roos EM. Joint injury causes knee osteoarthritis in young adults. *Curr Opin Rheumatol* 2005;17:195-200.
15. Papageorgiou CD, Gil JE, Kanamori A et al. The biomechanical interdependence between the anterior cruciate ligament replacement graft and the medial meniscus. *Am J Sports Med* 2001;29:226-31.
16. Butler RJ, Minick KI, Ferber R et al. Gait mechanics after ACL reconstruction: implications for the early onset of knee osteoarthritis. *Br J Sports Med* 2009;43:366-70.

For peer review only

17. Webster KE, Feller JA. The knee adduction moment in hamstring and patellar tendon anterior cruciate ligament reconstructed knees. *Knee Surg Sports Traumatol Arthrosc* 2012;20:2214-9.

18. Zabala ME, Favre J, Scanlan SF et al. Three-dimensional knee moments of ACL reconstructed and control subjects during gait, stair ascent, and stair descent. *J Biomech* 2013;46:515-20.

19. Miyazaki T, Wada M, Kawahara H et al. Dynamic load at baseline can predict radiographic disease progression in medial compartment knee osteoarthritis. *Ann Rheum Dis* 2002;61:617-22.

20. von Elm E, Altman DG, Egger M, et al; STROBE Initiative. The Strengthening the Reporting of Observational Studies in Epidemiology (STROBE)statement: guidelines for reporting observational studies. J Clin Epidemiol. 2008 Apr;61(4):344-9.

21. Roos EM, Roos HP, Lohmander LS et al. Knee Injury and Osteoarthritis Outcome Score (KOOS)— development of a self-administered outcome measure. *J Orthop Sports Phys Ther* 1998;28:88–96.

22. Tegner Y, Lysholm J. Rating systems in the evaluation of knee ligament injuries. Clin Orthop. 1985;198:43–49.

23. Cleather DJ, Bull AM. Influence of inverse dynamics methods on the calculation of inter-segmental moments in vertical jumping and weightlifting. *Biomed Eng Online* 2010;9:74

24. Cleather DI, Bull Am. Lower-extremity musculoskeletal geometry affects the calculation of patellofemoral forces in vertical jumping and weightlifting. *Proc Inst Mech Eng H* 2010;224:1073-1083

For peer review only - http://bmjopen.bmj.com/site/about/guidelines.xhtml

25. Hope, N., Duffell, L. D., and McGregor, A. H. Validation of a new model to calculate joint kinematics during gait. *European Society of Biomechanics Conference Proceedings* 1-7-2012.

26. Claes S, Hermie L, Verdonk R et al. Is osteoarthritis an inevitable consequence of anterior cruciate ligament reconstruction? A meta- analysis. *Knee Surg Sports Traumatol Arthrosc* 2013;21:1967-76.

27. Louboutin H, Debarge R, Richou J et al. Osteoarthritis in patients with anterior cruciate ligament rupture: a review of risk factors. *Knee* 2009;16:239-44.

28. Oiestad BE, Engebretsen L, Storheim K, et al. Knee osteoarthritis after anterior cruciate ligament injury: a systematic review. Am J Sports Med 2009;37:1434–1443

29. Monsch ED, Franz CO, Dean JC. The effects of gait strategy on metabolic rate and indicators of stability during downhill walking. *J Biomech* 2012;45:1928-33.

30. Mizner RL, Snyder-Mackler L. Altered loading during walking and sit-to-stand is affected by quadriceps weakness after total knee arthroplasty. *J Orthop Res* 2005;23:1083-90.

31. A.N. Lay, C.J. Hass, R.J. Gregor. The effects of sloped surfaces on locomotion: a kinematic and kinetic analysis. *Journal of Biomechanics* 2006;39:1621–1628

32. Gottschall JS, Nichols TR. Neuromuscular strategies for the transitions between level and hill surfaces during walking. *Philos Trans R Soc Lond B Biol Sci* 2011;366:1565-79.

33. Creaby MW, Bennell KL, Hunt MA. Gait differs between unilateral and bilateral knee osteoarthritis. *Arch Phys Med Rehabil* 2012;93(5):822-7.

For peer review only

34. Christiansen CL, Stevens-Lapsley JE. Weight-bearing asymmetry in relation to 378 measures of impairment and functional mobility for people with knee osteoarthritis. *Arch Phys Med Rehabil* 2010;91:1524-8.

35. Lohmander, L.S., Ostenberg, A., Englund, M. et al. High prevalence of knee osteoarthritis, pain, and functional limitations in female soccer players twelve years after anterior cruciate ligament injury. *Arthritis and Rheumatology* 2004;50:3145–3152.

36. Mündermann A, Dyrby CO, Hurwitz DE et al. Potential strategies to reduce medial compartment loading in patients with knee osteoarthritis of varying severity: reduced walking speed. *Arthritis Rheum* 2004;50:1172-8.

Figures

Fig. 1 Steel framed ramp covered in plywood, set an incline of 10 degrees.

Fig. 2 Peak adduction moments in **a)** level walking and **b)** slope walking for ACLR, ACLR +, ACLR – and control group. Asterisk indicates significance (p = 0.042).

Fig. 3, a) Peak flexion moment in all activities for ACLR, contralateral and control group. § represents decline gait to be significantly higher than all other activities (p<0.01). Asterisk indicates significance, p<0.05 **b)** Peak extension moment in all activities for ACLR, contralateral and control group. ~ represents decline gait to be significantly lower than normal and incline gait, p<0.05

For peer review only - http://bmjopen.bmj.com/site/about/guidelines.xhtml

Title: Knee moments of anterior cruciate ligament reconstructed and control participants during normal and inclined walking.

Authors: Raghav K Varma[1], Lynsey D Duffell[1], Dinesh Nathwani[1] & Alison H McGregor [1].

Affiliations: 1) MSK Lab, Imperial College London, UK

Corresponding Author:

Professor Alison McGregor

MSK Lab

Imperial College London

Floor 7, Laboratory Block

Charing Cross Hospital, Fulham Palace Road

London, W6 8RP

T: 020 3313 8834

F: 020 3313 8835

E: a.mcgregor@imperial.ac.uk

For peer review only

Word count: 3056

Key words: Anterior cruciate ligament reconstruction, inclined walkway, adduction moment and gait analysis.

Abstract

Objectives

Prior injury to the knee, particularly anterior cruciate ligament (ACL) injury is known to predispose to premature osteoarthritis (OA). The study sought to explore if there was a biomechanical rationale for this process by investigating changes in external knee moments between people with a history of ACL injury and uninjured subjects during walking; i) on different surface inclines and; ii) at different speeds. In addition we assessed functional differences between the groups.

Participants

Twelve subjects who had undergone ACL reconstruction (ACLR) and 12 volunteers with no history of knee trauma or injury were recruited into this study. Peak knee flexion and adduction moments were assessed during flat (normal and slow speed), uphill and downhill walking using an inclined walkway with an embedded Kistler Force plate, and a ten camera Vicon motion capture system. Knee osteoarthritis outcome score (KOOS) was used to assess function. MANOVA was used to examine statistical differences in gait and KOOS outcomes.

Results

No significant difference was observed in the peak knee adduction moment between ACLR and control participants however, in further analysis, MANOVA revealed that ACLR participants with an additional meniscal tear or collateral ligament damage (7 subjects) had a significantly higher adduction moment (0.33 ± 0.12 Nm/kg.m) when compared to those with isolated ACLR (5 subjects, 0.1 ± 0.057 Nm/kg.m) during gait at

For peer review only - http://bmjopen.bmj.com/site/about/guidelines.xhtml

their normal speed ($p<0.05$). A similar (non-significant) trend was seen during slow, uphill and downhill gait.

Conclusion

Subjects with an isolated ACLR had a reduced adductor moment rather an increased moment, thus questioning prior theories on OA development. In contrast those subjects who had sustained associated trauma to other key knee structures were observed to have an increased adduction moment. Additional injury concurrent with an ACL rupture may lead to a higher predisposition to osteoarthritis than isolated ACL deficiency alone.

Article summary

Strengths and limitations of this study

- To our knowledge this is the first report looking at external moments during inclined and declined walking for ACL reconstruction participants.
- In addition to looking into the external moments of the affected and unaffected knee, this study also looked at the effect of gait speed on external moments and differences in knee osteoarthritis outcome score (KOOS) for each group.
- This study provides a potential explanation for the disparity seen in previous studies looking into peak knee adduction moment in ACLR and matched control subjects.
- This study suggests that injuries to other key knee structures may play a bigger part in inducing osteoarthritis than ACL injury alone.

For peer review only

- One limitation to this study is the small sample size, in particular after dividing our ACLR group into ACLR + and ACLR – groups.

Introduction

Anterior Cruciate Ligament (ACL) injuries are common, exceeding 100,000 annual cases in the United States.(1) The majority are sports related injuries, and lead to knee instability as a result of increased anterior tibial translation and anterolateral rotation.(2) ACL reconstruction (ACLR) is the primary treatment for an ACL rupture and permits return to a range of high-level activities including sport. It is accepted that people with ACL injuries, including those who undergo surgical reconstruction, are prone to further knee degeneration(3) and early OA.(4,5) Lohmander et al. (2007) reviewed 127 publications and determined that the overall mean incidence of developing OA after an ACL injury with/without reconstruction to be over 50%(3) with the majority noting radiographic signs of OA 10 years after injury.(3) Gait biomechanics are considered to play a vital part in knee joint degeneration, (5, 6) with altered kinematics and kinetics changing the distribution of mechanical load on the knee. (7) This in turn is postulated to lead to cartilage wear(5 - 7) and eventually knee osteoarthritis.

There is consensus amongst researchers that ACL deficient patients employ different gait strategies.(8) In vivo studies have found reduced knee flexion,(5) increased internal tibial rotation(5, 9) and increased knee adduction moment(10) during level walking, to be the three main changes in external knee moments following an ACL rupture. Furthermore, research has indicated that ACL reconstruction does not restore normal knee mechanics(11). Berchuck et al. in 1990(12) noted reduced knee flexion during

For peer review only - http://bmjopen.bmj.com/site/about/guidelines.xhtml

normal gait, indicating a coping strategy termed quadriceps avoidance gait. Anterior displacement of tibia through the contraction of quadriceps is balanced by the ACL when the knee is at an angle of 0 to 45 degrees.(12) People with ACL rupture and/or reconstruction are found to have quadriceps activation deficits(13), which may be due to a central regulatory mechanism to avoid further joint damage by these muscle groups. Gait adaptations in the sagittal plane can lead to knee joint instability and ligament laxity.(14) This may result in osteoarthritis initiation and progression.(14)

High moments in frontal and transverse planes of the knee have been linked to OA.(5) ACLR has been shown to restore rotational stability,(9, 15) however high knee adduction moments (KAM) after reconstruction have been observed(16) but such changes are not universally agreed.(17, 18) This is of particular importance since a 1% increase in adduction moment at the knee is thought to increase the risk of knee OA by 6.5 times.(19) The discrepancies in previous studies may be due to different walking speeds, and higher KAM may only be evident during more challenging tasks. Hence in this study we aimed to gain a better understanding of peak knee moments in the frontal and sagittal plane during gait at different speeds and inclines. Our primary aim is to compare peak knee moments in the sagittal and frontal plane of ACLR participants to healthy controls on sloped surfaces, with a view to exploring the biomechanical basis for the observation that ACL injury predisposes to OA. Our secondary aim was to investigate the effect of speed on peak moments. Finally, we compared functional outcome scores between groups using the Knee injury and Osteoarthritis Outcome Score (KOOS).

For peer review only - http://bmjopen.bmj.com/site/about/guidelines.xhtml

Methods

This cross-sectional study explored peak knee moments between ACLR and healthy control subjects during slope walking. The study was approved by Imperial College Research Ethics Committee. We used the STROBE statement as a checklist for our observational study. (20)

A total number of 24 subjects participated in this study and written informed consent obtained, details are provided in Table 1. The ACLR inclusion criteria were; aged between 18 – 60 years, Body mass index (BMI) < 30kg/m^2, a complete, unilateral ACL rupture followed by a single bundle hamstring auto graft reconstruction that was performed at least one-year ago with no history of knee trauma or injury to their contralateral leg. Subjects who were unable to walk comfortably on a 10-degree incline walkway were also excluded. The control group did not have any muscular or neurological lower limb pathology and were matched to the ACLR subjects with respect to gender, activity, height, weight and their dominant leg (leg preference for kicking). All subject completed the Knee injury and Osteoarthritis Outcome Score (KOOS)(21). We measured the subject's activity levels using Tegner Activity scale (22).

A three-dimensional motion analysis system (Vicon MX™ T-20 System, Vicon, Oxford) was used to collect kinematic data for normal, slow, upslope and downslope gait. This software used 10 motion capture cameras to pick up 35 reflective markers at a sampling rate of 100Hz. The reflective markers were placed bilaterally on the head of 2nd metatarsal, head of 5th metatarsal, head of talus, calcaneal tuberosity, medial and lateral malleolus, medial and lateral femoral epicondyle, anterior superior iliac spine, posterior superior iliac spine, acromion and one single marker on the manubrium. Marker clusters

For peer review only - http://bmjopen.bmj.com/site/about/guidelines.xhtml

(3 reflective markers on each) were affixed bilaterally to the calf and thigh. Kinetic data (ground reaction force) were collected using portable force plates (Kistler Instruments AG, Winterthur, Switzerland) at a sampling rate of 1000 Hz and was synchronised with the camera data.

The subjects were asked to walk barefoot. A 5 minute self-directed warm up allowed the subjects to familiarise themselves with each task.

A 7-meter long walkway was used, 2.5-meters of which could be raised to form a ramp, at an incline of 10 degrees. It was constructed from a steel frame and covered with plywood, with one portable force plate embedded in the centre (Figure 1). Participants were asked to walk at a self-selected pace both uphill and downhill. The ramp was then removed to create a level walkway. All the participants were asked to walk at a self-selected pace and at a pace, which they consider to be slow. Each task was repeated until both feet made complete contact with the middle of the force plate at least three times.

All data was time normalised to one gait cycle. The data was analysed from the stance phase of the gait cycle; when the ground reaction force reached more than 40N (heel strike) to when it dropped to less than 40N (toe off). A fourth order Butterworth Filter at a cut off (12 Hz) was used to reduce noise. Joint angles and moments were calculated from the position of the reflective markers and the ground reaction force data using a custom model written in body builder software.(23-25) Peak moments in the sagittal and frontal planes of the knee were extracted using MATLAB (R2013b) software.

For peer review only - http://bmjopen.bmj.com/site/about/guidelines.xhtml

Unpaired Student's t-tests were used to determine any significant differences in demographics between ACLR and control group. MANOVA test was used to calculate significant differences in all other parameters. Tukey HSD approach was used to establish significance, with the alpha value set at 0.05. All statistics were carried out using SPSS version 22.

Results

12 ACLR participants (9 male and 3 female) and 12 control (9 male and 3 female) were recruited for this study. There were no significant differences between groups in age, height, weight and Tegner Activity Scale (Table 1). The mean time since reconstruction surgery was 4 years and 6 months.

Table 1 Subject characteristics, activity level and time since surgery. 12 participants in ACLR group and 12 in control group

	ACLR (SD)	Control (SD)	Unpaired T-test
Age (yr)	30.5 (8.68)	24.8 (8.81)	P = 0.125
Height (m)	1.76 (0.13)	1.73 (0.11)	P = 0.547
Weight (kg)	75 (11.13)	71.6 (11.2)	P = 0.464
Tegner Activity Scale	6.25 (1.82)	6.08 (1.93)	P = 0.826
Time since surgery (yr)	4.5 (3.5)	NA	

ACLR, Anterior cruciate ligament reconstruction

We further divided our ACLR group into two; subjects that had additional cartilage, meniscus or ligament damage in their ACLR leg (ACLR + group; 7 subjects) and subjects with isolated ACL injuries (ACLR – group; 5 subjects). The additional knee injuries to the ACL rupture are meniscal tear (3 participants), cartilage damage (3 participants) and torn MCL (1 participant).

For peer review only - http://bmjopen.bmj.com/site/about/guidelines.xhtml

No statistically significant differences were found in peak knee adduction moment between ACLR and control participants during uphill and downhill gait, and during gait at normal and slow walking speeds on a flat surface (Figure 2). Further analysis revealed that ACLR participants with meniscal tear, cartilage damage or medial collateral ligament damage (ACLR +) had significantly higher knee adduction moment (0.33 ± 0.12 Nm/kg.m) during gait on a flat surface at a normal walking speed compared to those participants with an isolated ACL injury (ACLR -, 0.1 ± 0.057 Nm/kg.m), p = 0.042 (Figure 2).

This was not the case for data collected in the sagittal plane. There was a tendency for the contralateral (unaffected) knee of ACLR participants to show a higher mean knee flexion moment in all activities compared to ACLR affected knees and control knees (Figure 3). The difference was not found to be statistically significant.

Table 2 shows that there were no significant differences in gait speed in normal or slow walking between any groups. The control group had significantly higher scores in all of the KOOS domains apart from activities of daily life compared to the ACLR group (Table 3). No significant KOOS differences were seen between ACLR + and ACLR – groups.

Table 2 Gait speed during normal and slow, level walking tasks.

	ACLR	Control	ACLR +	ACLR -	P = value
Gait normal speed	1.17 (0.13)	1.20 (0.11)	1.18 (0.15)	1.16 (0.11)	P = 0.940
Gait slow speed	0.76 (0.13)	0.75 (0.11)	0.78 (0.16)	0.74 (0.09)	P = 0.885

ACLR, Anterior cruciate ligament reconstruction; ACLR +, subjects with other knee injuries in their ACLR leg; ACLR -, subjects with isolated ACL injuries.
Data are mean (SD).

For peer review only - http://bmjopen.bmj.com/site/about/guidelines.xhtml

Table 3 Knee osteoarthritis outcome score (KOOS) with standard deviation (SD) for each domain recorded for each group.

KOOS Outcome	ACLR (SD) (n=12)	Control (SD) (n=12)	ACLR + (SD) (n=7)	ACLR - (SD) (n=5)	Significant values
Pain	88.4 (9.32)	99.1 (3.2)	87.5 (8.83)	89.4 (10.8)	Control vs ACLR: P = 0.010 Control vs ACLR(+): P = 0.019
Symptoms	83.1 (11.4)	98.2 (3.19)	85.1 (12.7)	80.7 (10.6)	Control vs all other groups: P < 0.05
Activities of daily life	96.3 (5.63)	100 (0)	98 (3)	94.4 (7.7)	No significant differences
Sport and recreation	83.8 (16.9)	99.6 (1.4)	89.1 (7.4)	77.4 (23)	Control vs ACLR: P = 0.006 Control vs ACLR(-): P = .003
Knee related QOL	64.5 (23.2)	100 (0)	64.6 (23.3)	70 (26.7)	Control vs all other groups: P < 0.05

ACLR, Anterior cruciate ligament reconstruction; ACLR +, subjects with other knee injuries in their ACLR leg; ACLR -, subjects with isolated ACL injuries.
Data are mean (SD).

Discussion

In the frontal plane we found no statistically significant differences between our ACLR participants and controls. However ACLR subjects who had sustained associated trauma to other key knee structures (meniscal, collateral ligament and chondral damage) were observed to have a higher adduction moment during gait on a flat surface at normal walking speed when compared to subjects with isolated ACLR. In the sagittal

For peer review only - http://bmjopen.bmj.com/site/about/guidelines.xhtml

plane there was a tendency for ACLR participants to have higher peak knee flexion moment in their contralateral leg during all activities.

Previous studies that have investigated peak knee adduction moment in ACLR and matched control subjects have provided mixed results.(16,17,18) We investigated similar and more challenging gait set-ups (by altering incline), as well as the effects of differences in walking speed, in order to explore the biomechanical basis for the observation that ACL injury predisposes to OA. Our data showed no significant differences in KAM between our ACLR participants and control subjects under all conditions. This indicates that providing more challenging gait set-ups such as sloped walking, where a higher range of motion in the sagittal plane is required, does not emphasize differences between ACLR and control subjects. Based on our findings, the discrepancies in previous studies appear not to be related to the difficulty of the task or differences in walking speed.

ACL injury is often accompanied by other knee injuries.(26) Prevalence of associated meniscal damage and chondral lesions at the time of ACL injury can be as high as 65% and 23% respectively.(27) Associated knee injuries are thought to increase the incidence of OA from 0 – 13% in isolated ACL injury to 21 – 48%.(28) This may be because key knee structures such as menisci prevent cartilage wear by distributing loads and functioning as a shock absorber.(14) Our results suggest that people with ACLR injuries with associated knee injuries experience higher knee adduction moments than people with isolated ACLR injuries.

For peer review only - http://bmjopen.bmj.com/site/about/guidelines.xhtml

Of the three previous studies that have looked at peak KAM in ACLR and matched control subjects, Butler et al. found the ACLR group to have a higher peak KAM compared to controls.(16) This was also seen by our ACLR + group. In the studies that followed, Webster et al. and Zabala et al. found the ACLR group to have a reduced peak knee adduction moment to controls.(17, 18) This was seen by our ACLR – group (Figure 2). This disparity in studies may therefore be a consequence of different exclusion criteria for ACLR subjects. Butler et al. did not exclude ACLR subjects with other knee injuries,(16) while the other two studies excluded subjects with ligament damage(17, 18) and also those with >25% of menisci loss.(18) We suggest that associated knee injuries are related to increased knee adduction moments in ACLR participants.

We found the difference between peak KAM in ACLR + and ACLR – to be statistically significant only during normal gait. We expected the difference to be higher during sloped walking, as it is more challenging than level walking. Change in terrain, muscle weakness, gait deficit and balance deficit are primary risk factors for falling.(29, 30) This suggests that ACLR participants need to adopt a conservative gait strategy while walking on a sloped surface to ensure safety. During challenging tasks such as downhill walking healthy participants increased their metabolic activity and implemented a conservative gait strategy to reduce the risk of falling.(29) This principle may also be applied by ACLR participants to ensure safety.

In the sagittal plane, no statistically significant differences were observed between ACLR, ACLR +, ACLR – and control group in peak knee flexion and extension moments. Both uphill and downhill walking require greater use of quadriceps muscle than on a level walkway.(30, 31) Two years after an ACLR surgery, differences in quadriceps

For peer review only - http://bmjopen.bmj.com/site/about/guidelines.xhtml

strength between limbs are no longer seen.(29) All of the ACLR subjects had undergone reconstruction at least 1 year before taking part in this study, with an average of 4.5 years. This indicates that all subjects may have had sufficient time to restore their quadriceps strength. Even though sagittal instability is thought to increase joint loads and lead to joint failure,(14, 32) it may not play a significant role in OA induction and progression after reconstruction.

An unexpected finding was the tendency for the contralateral knees of ACLR participants to have higher peak knee flexion and lower peak knee extension moment compared to the ACLR and control knee in all activities. This may be an adaptation to reduce loading on their ACLR knee. Patients with advanced knee OA also display this adaptation to reduce loading on their injured leg.(33) Even though this may present as a mechanism to slow the progression of OA, there are harmful implications associated with the contralateral leg. Weight bearing asymmetry may induce OA in the contralateral leg;(34) 37% (24/65 female patients) showed signs of radiographic OA in their contralateral leg, 12 years after ACL reconstruction.(35) This suggests that in this population unilateral injury changes joint function bilaterally.

In addition to our primary investigations we also investigated differences in KOOS and walking speed. There was no significant difference in KOOS or gait speed between ACLR + and ACLR –. This indicates that high peak knee adduction moment in the injured leg does not affect our subjects' pain outcome, symptoms, activities of daily life, sport and recreation, knee related quality of life and gait speed. Thereby patient reported outcome measures and gait speed might not provide the clinician with any information about different gait adaptations.

For peer review only - http://bmjopen.bmj.com/site/about/guidelines.xhtml

For peer review only

Mundermann et al (2004) found a 10.2% reduction in maximum knee adduction moment when people with less severe OA reduced their walking speed from 1.2 meters/second to 0.8 meters per second.(36) However in the current study the difference in peak knee moments between normal (1.17 m/s) and slow gait speed (0.76 m/s) were not statistically significant. This may be due to our small sample size.

It is important to note that different knee injuries, rehabilitation protocols and time between injury and reconstruction are all thought to influence joint moments.(18) These are limitations that should be considered when examining the results presented here. Additionally our participants were not recruited straight after their ACLR, some participants may have had further injury or pathological changes within the joint since the reconstruction. Another limitation was our small sample size, in particular after dividing our ACLR group into ACLR + and ACLR – groups.

The ramp was set at an incline of 10 degrees because the transition from a level to slope walking strategy is thought to be around 5.5 degrees(31) and after the incline of 10 degrees no kinematic differences are seen in healthy participants.(31)

With regards to Vicon Motion capture system different skin marker placement and skin motion artifacts are thought to increase error.(6) We tried to reduce the effects of different skin marker placement by having only one researcher place all markers on each individual. In addition we used a model that used clusters to reduce the effects of skin motion.(25)

For peer review only - http://bmjopen.bmj.com/site/about/guidelines.xhtml

Conclusion

In conclusion, this study found no significant differences in peak moments in the frontal and sagittal plane during level and sloped walking for ACLR compared with control participants. However, we noted that individuals who have other knee injuries associated with their ACLR knee exhibit higher peak adduction moments during level walking at their normal speed. This suggests that injuries to other key knee structures may play a bigger part in inducing OA than ACL injury alone, although this requires further investigation with a larger sample size. Our data also suggest that the contralateral knee appears to be functioning in such a way to reduce high moments in the ACLR knees, which may be relevant in the risk of OA development in both knees. These findings warrant a longitudinal study comparing the knee adduction moment between isolated ACLR injury and ACLR with additional knee injuries and the prevalence of premature OA.

Data sharing - no additional data available

Acknowledgements - The authors acknowledge support from Mr. R. Weinert-Aplin for the facilitation of the instrumented inclined walkway and conversion of the output data.

Competing interests - None

Funding - The authors are also thankful for the funding provided by the Wellcome Trust and the EPSRC.

Contributorship Statement - All authors helped with the study design. Raghav K Varma carried out data collection, statistical analysis and analysis of the data produced. He also drafted and revised the paper. He is guarantor. Lynsey D Duffell attained ethical

For peer review only - http://bmjopen.bmj.com/site/about/guidelines.xhtml

approval, determined the methodology, helped with data analysis and drafted and revised the paper. Dinesh Nathwani helped with data collection and drafted and revised the paper. Alison H McGregor monitored the data collection for the whole trial, provided the data collection tools and drafted and revised the paper. Robert Weinert-Aplin designed the data collection tool.

References

1. Lyman S, Koulouvaris P, Sherman S et al. Epidemiology of anterior cruciate ligament reconstruction: trends, readmissions, and subsequent knee surgery. *J Bone Joint Surg Am* 2009;91:2321-8.
2. Matsumoto H, Suda Y, Otani T et al. Roles of the anterior cruciate ligament and the medial collateral ligament in preventing valgus instability. *J Orthop Sci* 2001;6:28-32.
3. Lohmander LS, Englund PM, Dahl LL et al. The long-term consequence of anterior cruciate ligament and meniscus injuries: osteoarthritis. *Am J Sports Med* 2007;35:1756-69.
4. Porat A, Roos EM, Roos H. High prevalence of osteoarthritis 14 years after an anterior cruciate ligament tear in male soccer players: a study of radiographic and patient relevant outcomes. *Ann Rheum Dis* 2004;63:269-73.
5. Andriacchi TP, Mündermann A. The role of ambulatory mechanics in the initiation and progression of knee osteoarthritis. *Curr Opin Rheumatol* 2006;18:514-8.
6. Stergiou N, Ristanis S, Moraiti C et al. Tibial rotation in anterior cruciate ligament (ACL)-deficient and ACL-reconstructed knees: a theoretical proposition for the development of osteoarthritis. *Sports Med* 2007;37:601-13.

For peer review only

For peer review only - http://bmjopen.bmj.com/site/about/guidelines.xhtml

7. Andriacchi TP, Mündermann A, Smith RL et al. A framework for the in vivo pathomechanics of osteoarthritis at the knee. *Ann Biomed Eng* 2004;32:447-57.

8. Hart JM, Ko JW, Konold T et al. Sagittal plane knee joint moments following anterior cruciate ligament injury and reconstruction: a systematic review. *Clin Biomech* 2010;25:277-83.

9. Georgoulis AD, Papadonikolakis A, Papageorgiou CD et al. Three-dimensional tibiofemoral kinematics of the anterior cruciate ligament-deficient and reconstructed knee during walking. *Am J Sports Med* 2003;31:75-9.

10. Noyes FR, Schipplein OD, Andriacchi TP et al. The anterior cruciate ligament-deficient knee with varus alignment. An analysis of gait adaptations and dynamic joint loadings. *Am J Sports Med* 1992;20:707-16.

11. Gokeler A, Benjaminse A, van Eck CF, et al. Return of normal gait as an outcome measurement in acl reconstructed patients. A systematic review. Int J Sports Phys Ther. 2013 Aug;8(4):441-51.

12. Berchuck M, Andriacchi TP, Bach BR et al. Gait adaptations by patients who have a deficient anterior cruciate ligament. *J Bone Joint Surg Am* 1990;72:871-7.

13. Urbach D, Nebelung W, Becker R et al. Effects of reconstruction of the anterior cruciate ligament on voluntary activation of quadriceps femoris a prospective twitch interpolation study. *J Bone Joint Surg Br* 2001;83:1104-10.

14. Roos EM. Joint injury causes knee osteoarthritis in young adults. *Curr Opin Rheumatol* 2005;17:195-200.

15. Papageorgiou CD, Gil JE, Kanamori A et al. The biomechanical interdependence between the anterior cruciate ligament replacement graft and the medial meniscus. *Am J Sports Med* 2001;29:226-31.

For peer review only - http://bmjopen.bmj.com/site/about/guidelines.xhtml

16. Butler RJ, Minick KI, Ferber R et al. Gait mechanics after ACL reconstruction: implications for the early onset of knee osteoarthritis. *Br J Sports Med* 2009;43:366-70.

17. Webster KE, Feller JA. The knee adduction moment in hamstring and patellar tendon anterior cruciate ligament reconstructed knees. *Knee Surg Sports Traumatol Arthrosc* 2012;20:2214-9.

18. Zabala ME, Favre J, Scanlan SF et al. Three-dimensional knee moments of ACL reconstructed and control subjects during gait, stair ascent, and stair descent. *J Biomech* 2013;46:515-20.

19. Miyazaki T, Wada M, Kawahara H et al. Dynamic load at baseline can predict radiographic disease progression in medial compartment knee osteoarthritis. *Ann Rheum Dis* 2002;61:617-22.

20. von Elm E, Altman DG, Egger M, Pocock SJ, Gøtzsche PC, Vandenbroucke JP; STROBE Initiative. The Strengthening the Reporting of Observational Studies in Epidemiology (STROBE)statement: guidelines for reporting observational studies. J Clin Epidemiol. 2008 Apr;61(4):344-9.

21. Roos EM, Roos HP, Lohmander LS et al. Knee Injury and Osteoarthritis Outcome Score (KOOS)— development of a self-administered outcome measure. *J Orthop Sports Phys Ther* 1998;28:88–96.

22. Tegner Y, Lysholm J. Rating systems in the evaluation of knee ligament injuries. Clin Orthop. 1985;198:43–49.

23. Cleather DJ, Bull AM Influence of inverse dynamics methods on the calculation of inter-segmental moments in vertical jumping and weightlifting. *Biomed Eng Online* 2010;9:74

For peer review only

For peer review only - http://bmjopen.bmj.com/site/about/guidelines.xhtml

24. Cleather DI, Bull AM Lower-extremity musculoskeletal geometry affects the calculation of patellofemoral forces in vertical jumping and weightlifting. *Proc Inst Mech Eng H* 2010;224:1073-1083

25. Hope, N., Duffell, L. D., and McGregor, A. H. Validation of a new model to calculate joint kinematics during gait. *European Society of Biomechanics Conference Proceedings* 1-7-2012.

26. Claes S, Hermie L, Verdonk R et al. Is osteoarthritis an inevitable consequence of anterior cruciate ligament reconstruction? A meta- analysis. *Knee Surg Sports Traumatol Arthrosc* 2013;21:1967-76.

27. Louboutin H, Debarge R, Richou J et al. Osteoarthritis in patients with anterior cruciate ligament rupture: a review of risk factors. *Knee* 2009;16:239-44.

28. Oiestad BE, Engebretsen L, Storheim K, Risberg MA. Knee osteoarthritis after anterior cruciate ligament injury: a systematic review. Am J Sports Med 2009;37:1434–1443

29. Monsch ED, Franz CO, Dean JC. The effects of gait strategy on metabolic rate and indicators of stability during downhill walking. *J Biomech* 2012;45:1928-33.

30. Mizner RL, Snyder-Mackler L. Altered loading during walking and sit-to-stand is affected by quadriceps weakness after total knee arthroplasty. *J Orthop Res* 2005;23:1083-90.

31. A.N. Lay, C.J. Hass, R.J. Gregor. The effects of sloped surfaces on locomotion: a kinematic and kinetic analysis. *Journal of Biomechanics* 2006;39:1621–1628

32. Gottschall JS, Nichols TR. Neuromuscular strategies for the transitions between level and hill surfaces during walking. *Philos Trans R Soc Lond B Biol Sci* 2011;366:1565-79.

For peer review only

33. Creaby MW, Bennell KL, Hunt MA. Gait differs between unilateral and bilateral knee osteoarthritis. *Arch Phys Med Rehabil* 2012;93(5):822-7.

34. Christiansen CL, Stevens-Lapsley JE. Weight-bearing asymmetry in relation to measures of impairment and functional mobility for people with knee osteoarthritis. *Arch Phys Med Rehabil* 2010;91:1524-8.

35. Lohmander, L.S., Ostenberg, A., Englund, M. et al. High prevalence of knee osteoarthritis, pain, and functional limitations in female soccer players twelve years after anterior cruciate ligament injury. *Arthritis and Rheumatology* 2004;50:3145–3152.

36. Mündermann A, Dyrby CO, Hurwitz DE et al. Potential strategies to reduce medial compartment loading in patients with knee osteoarthritis of varying severity: reduced walking speed. *Arthritis Rheum* 2004;50:1172-8.

Figures

Fig. 1 Steel framed ramp covered in plywood, set an incline of 10 degrees.

Fig. 2 Peak adduction moments in **a)** level walking and **b)** slope walking for ACLR, ACLR +, ACLR – and control group. Asterisk indicates significance (p = 0.042).

Fig. 3, a) Peak flexion moment in all activities for ACLR, contralateral and control group. § represents decline gait to be significantly higher than all other activities (p<0.01). Asterisk indicates significance, p<0.05 **b)** Peak extension moment in all activities for ACLR, contralateral and control group. ~ represents decline gait to be significantly lower than normal and incline gait, p<0.05

For peer review only

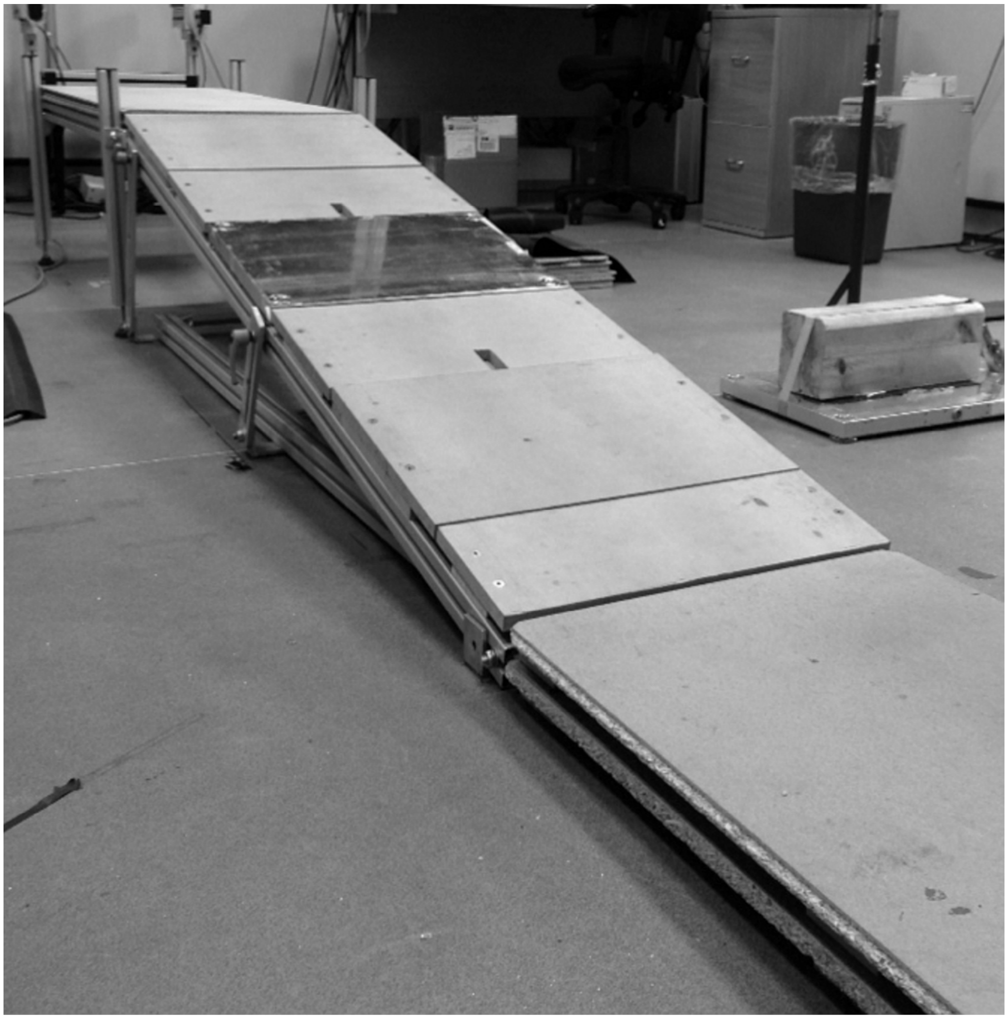

Steel framed ramp covered in plywood, set an incline of 10 degrees.
90x91mm (300 x 300 DPI)

For peer review only - http://bmjopen.bmj.com/site/about/guidelines.xhtml

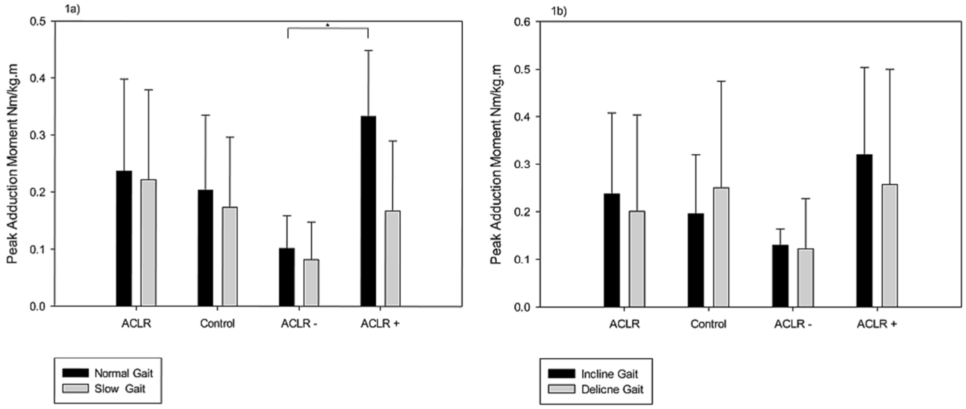

Peak adduction moments in a) level walking and b) slope walking for ACLR, ACLR +, ACLR – and control group. Asterisk indicates significance ($p = 0.042$).
90x42mm (300 x 300 DPI)

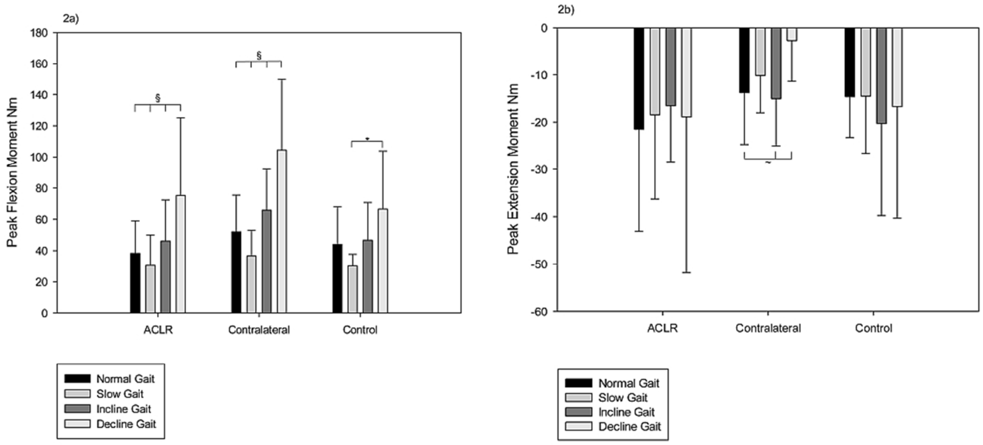

a) Peak flexion moment in all activities for ACLR, contralateral and control group. § represents decline gait to be significantly higher than all other activities (p<0.01). Asterisk indicates significance, p<0.05 b) Peak extension moment in all activities for ACLR, contralateral and control group. ~ represents decline gait to be significantly lower than normal and incline gait, p<0.05
90x44mm (300 x 300 DPI)

review only

STROBE Statement—checklist of items that should be included in reports of observational studies

	Item No	Recommendation
**Title and abstract**	1	(*a*) Indicate the study's design with a commonly used term in the title or the abstract
		(*b*) Provide in the abstract an informative and balanced summary of what was done and what was found
**Introduction**		
Background/rationale	2	Explain the scientific background and rationale for the investigation being reported
Objectives	3	State specific objectives, including any prespecified hypotheses
**Methods**		
Study design	4	Present key elements of study design early in the paper
Setting	5	Describe the setting, locations, and relevant dates, including periods of recruitment, exposure, follow-up, and data collection
Participants	6	(*a*) *Cohort study*—Give the eligibility criteria, and the sources and methods of selection of participants. Describe methods of follow-up<br>*Case-control study*—Give the eligibility criteria, and the sources and methods of case ascertainment and control selection. Give the rationale for the choice of cases and controls<br>*Cross-sectional study*—Give the eligibility criteria, and the sources and methods of selection of participants
		(*b*) *Cohort study*—For matched studies, give matching criteria and number of exposed and unexposed<br>*Case-control study*—For matched studies, give matching criteria and the number of controls per case
Variables	7	Clearly define all outcomes, exposures, predictors, potential confounders, and effect modifiers. Give diagnostic criteria, if applicable
Data sources/ measurement	8*	For each variable of interest, give sources of data and details of methods of assessment (measurement). Describe comparability of assessment methods if there is more than one group
Bias	9	Describe any efforts to address potential sources of bias
Study size	10	Explain how the study size was arrived at
Quantitative variables	11	Explain how quantitative variables were handled in the analyses. If applicable, describe which groupings were chosen and why
Statistical methods	12	(*a*) Describe all statistical methods, including those used to control for confounding
		(*b*) Describe any methods used to examine subgroups and interactions
		(*c*) Explain how missing data were addressed
		(*d*) *Cohort study*—If applicable, explain how loss to follow-up was addressed<br>*Case-control study*—If applicable, explain how matching of cases and controls was addressed<br>*Cross-sectional study*—If applicable, describe analytical methods taking account of sampling strategy
		(*e*) Describe any sensitivity analyses

Continued on next page

For peer review only

**Results**		
Participants	13*	(a) Report numbers of individuals at each stage of study—eg numbers potentially eligible, examined for eligibility, confirmed eligible, included in the study, completing follow-up, and analysed
		(b) Give reasons for non-participation at each stage
		(c) Consider use of a flow diagram
Descriptive data	14*	(a) Give characteristics of study participants (eg demographic, clinical, social) and information on exposures and potential confounders
		(b) Indicate number of participants with missing data for each variable of interest
		(c) *Cohort study*—Summarise follow-up time (eg, average and total amount)
Outcome data	15*	*Cohort study*—Report numbers of outcome events or summary measures over time
		Case-control study—Report numbers in each exposure category, or summary measures of exposure
		Cross-sectional study—Report numbers of outcome events or summary measures
Main results	16	(*a*) Give unadjusted estimates and, if applicable, confounder-adjusted estimates and their precision (eg, 95% confidence interval). Make clear which confounders were adjusted for and why they were included
		(*b*) Report category boundaries when continuous variables were categorized
		(*c*) If relevant, consider translating estimates of relative risk into absolute risk for a meaningful time period
Other analyses	17	Report other analyses done—eg analyses of subgroups and interactions, and sensitivity analyses
**Discussion**		
Key results	18	Summarise key results with reference to study objectives
Limitations	19	Discuss limitations of the study, taking into account sources of potential bias or imprecision. Discuss both direction and magnitude of any potential bias
Interpretation	20	Give a cautious overall interpretation of results considering objectives, limitations, multiplicity of analyses, results from similar studies, and other relevant evidence
Generalisability	21	Discuss the generalisability (external validity) of the study results
**Other information**		
Funding	22	Give the source of funding and the role of the funders for the present study and, if applicable, for the original study on which the present article is based

*Give information separately for cases and controls in case-control studies and, if applicable, for exposed and unexposed groups in cohort and cross-sectional studies.

Note: An Explanation and Elaboration article discusses each checklist item and gives methodological background and published examples of transparent reporting. The STROBE checklist is best used in conjunction with this article (freely available on the Web sites of PLoS Medicine at http://www.plosmedicine.org/, Annals of Internal Medicine at http://www.annals.org/, and Epidemiology at http://www.epidem.com/). Information on the STROBE Initiative is available at www.strobe-statement.org.

For peer review only
